# Insights from ^125^Te and ^57^Fe nuclear resonance vibrational spectroscopy: a [4Fe–4Te] cluster from two points of view†
†Electronic supplementary information (ESI) available: Supplementary Tables S1–S4, Fig. S1–S6, Materials and methods, atomic Cartesian coordinates, and animated vibrational modes. CCDC 1908709. For ESI and crystallographic data in CIF or other electronic format see DOI: 10.1039/c9sc02025j


**DOI:** 10.1039/c9sc02025j

**Published:** 2019-06-24

**Authors:** Florian Wittkamp, Nakul Mishra, Hongxin Wang, Hans-Christian Wille, René Steinbrügge, Martin Kaupp, Stephen P. Cramer, Ulf-Peter Apfel, Vladimir Pelmenschikov

**Affiliations:** a Department of Chemistry and Biochemistry , Inorganic Chemistry I , Ruhr-Universität Bochum , Universitätsstraße 150 , 44801 Bochum , Germany . Email: ulf.apfel@rub.de; b Department of Chemistry , University of California , Davis, One Shields Avenue , Davis , California 95616 , USA . Email: spjcramer@ucdavis.edu; c Deutsches Elektronen-Synchrotron DESY , Notkestraße 85 , 22607 Hamburg , Germany; d Institute of Chemistry , Theoretical Chemistry/Quantum Chemistry , Technical University of Berlin , Sekr. C7, Straße des 17. Juni 135 , 10623 Berlin , Germany . Email: pelmentschikov@tu-berlin.de; e Fraunhofer UMSICHT , Osterfelder Straße 3 , 46047 Oberhausen , Germany

## Abstract

Iron–sulfur clusters are common building blocks for electron transport and active sites of metalloproteins. Their comprehensive investigation is crucial for understanding these enzymes, which play important roles in modern biomimetic catalysis and biotechnology applications. We address this issue by utilizing (Et_4_N)_3_[Fe_4_Te_4_(SPh)_4_], a tellurium modified version of a conventional reduced [4Fe–4S]^+^ cluster, and performed both ^57^Fe- and ^125^Te-NRVS to reveal its characteristic vibrational features. Our analysis exposed major differences in the resulting ^57^Fe spectrum profile as compared to that of the respective [4Fe–4S] cluster, and between the ^57^Fe and ^125^Te profiles. DFT calculations are applied to rationalize structural, electronic, vibrational, and redox-dependent properties of the [4Fe–4Te]^+^ core. We herein highlight the potential of sulfur/tellurium exchange as a method to isolate the iron-only motion in enzymatic systems.

## Abstract 2

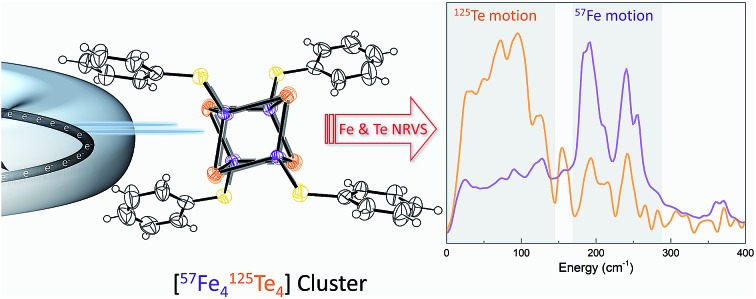
Can sulfur-to-tellurium exchange serve as a method to understand iron–sulfur clusters of enzymatic systems?

## Introduction

Tellurium is a component of many materials of current scientific and technological interest, including thermoelectrics such as PbTe-based materials,^1^,^2^ phase change materials for data storage such as Ge_2_Sb_2_Te_5_,^3^ and superconductors such as Fe_1+*y*_Te_1–*x*_Se_*x*_ and CdTe solar cells.^4^,^5^ Although Te is not an essential trace element in biological systems, certain Te compounds can be bioaccumulated and/or metabolized and some of them have shown or have been proposed to have valuable antibiotic or anticancer applications.^6^ This contrasts with the lighter chalcogens, S and Se, where S is ubiquitous in most living systems, and Se plays an essential role as selenocysteine.^7^–^9^ Only in the late 1980's, storage and transport of semimetal tellurium was observed in biological systems.^10^ When the tellurium-tolerant fungi *Penicillium chrysogenum* was fed with sodium tellurite containing media, Te incorporation was observed to afford telluro-cysteine and -methionine.^10^

[4Fe–4S] clusters are one of the most common bioinorganic prosthetic groups, playing crucial roles in many biological processes, such as electron transfer,^11^,^12^ O_2_ and NO-sensing, or even as active sites in radical SAM enzymes.^13^–^17^ The role of sulfur atoms in a cofactor is commonly not specifically elucidated, since sulfur spectroscopy of enzymes remains a challenging task due to the sulfur-rich environment of the peptide backbone. Current studies on [4Fe–4S] cluster compounds (or Fe–S interactions in general) are limited to probing the iron sites. Notably, the naturally low abundance of tellurium within an organism makes this element a unique tool to investigate specific processes by following the trace of tellurium, *e.g.,* substituting sulfur(s) at specified locations in biological samples and evaluating the metal–sulfur interaction(s) using difference spectra.

Nuclear resonance vibrational spectroscopy (NRVS),^18^ also known as nuclear inelastic scattering (NIS), is a relatively new synchrotron-based technique that yields vibrational spectra from nuclear transitions of an appropriate Mössbauer isotope. The key feature of this technique is that it is only sensitive to the motion of the selected isotope along the direction of the incident X-ray beam. For example, in the case of ^57^Fe-enriched proteins, this allows one to select exclusively the normal modes involving the ^57^Fe nuclei in a background of thousands of other vibrations involving the protein matrix. ^57^Fe-NRVS has already been used to investigate a wide variety of Fe-containing proteins, including rubredoxins, ferredoxins,^19^,^20^ heme proteins,^21^ hydrogenases,^22^,^23^ dioxygenases,^24^ NO sensors, and nitrogenase.^25^

In 2010, NRVS was first applied to ^125^Te with its nuclear resonance at 35.49 keV.^26^ Thanks to the development of sapphire backscattering monochromators,^27^ spectra with 1.1 meV (<9 cm^–1^) resolution have been obtained for elemental Te,^27^ Bi_2_Te_3_ and Sb_2_Te_3_,^28^–^30^ as well as GeTe, SnTe, and PbTe.^31^^125^Te presents a unique opportunity for bioinorganic NRVS studies thanks to the feasibility of Te for S substitution. Te can replace bridging S in Fe–S clusters, and it can also replace S to yield tellurocysteine and telluromethionine.

Despite growing popularity for materials science, there have been no reports of ^125^Te-NRVS of biologically relevant complexes to date. In this study we investigate the feasibility of bioinorganic ^125^Te-NRVS studies using tellurium modification of a well-known [4Fe–4S] cluster, resulting in a ^57^Fe and ^125^Te enriched (Et_4_N)_3_[Fe_4_Te_4_(SPh)_4_] model compound. Dual isotopic labelling allows for deeper insights into the dynamics of the cluster; since certain modes can be strong in one spectrum and weak or absent in the partner spectrum. Here, the unique ability to combine NRVS measurements of both ^57^Fe and ^125^Te in the same sample offers added assessment by density functional theory (DFT) which was applied to analyze the vibrational modes of the [4^57^Fe–4^125^Te] core in a greater detail.

Herein, we provide a benchmark study to reveal the potential of ^125^Te-NRVS and a combination of isotopic labelling of both iron and tellurium as a probe in electron transport processes in future *in vivo* studies within enzymes' Fe–S systems, such as [FeFe]-hydrogenases and CO-dehydrogenases.

## Results and discussion

### Synthesis

**Scheme 1:**
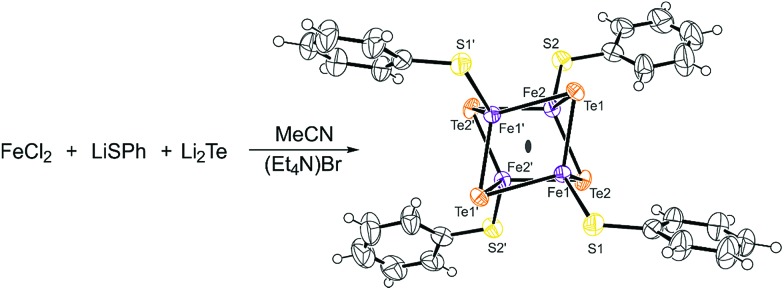
Schematic representation of the synthesis and ORTEP molecular plot of (Et_4_N)_3_[Fe_4_Te_4_(SPh)_4_]. The probability level is 50%. Counter ions are omitted for clarity.

(Et_4_N)_3_[^57^Fe_4_^125^Te_4_(SPh)_4_] = **1** was synthesized combining the synthetic procedures reported by Midollini^32^ and Haase,^33^*via* the reaction of FeCl_2_, LiSPh and Li_2_Te to obtain black needle-shaped crystals (Scheme 1).

The black crystals were analyzed by ^1^H NMR spectroscopy and X-ray diffractometry. The proton NMR spectrum (Fig. S1†) shows three paramagnetic signals at 13.5 ppm (*meta*), –3.1 ppm (*ortho*) and –4.1 ppm (*para*) in a 2 : 2 : 1 ratio and are assigned to the aromatic protons of the anionic cluster. In addition, two diamagnetic signals at 3.04 ppm and 1.35 ppm in a ratio of 2 : 3 are observed which can be assigned to the tetraethyl ammonium counter ions. The overall spectrum closely resembles the spectrum presented by Midollini *et al.*^32^

The obtained crystals were of decent quality (*R* = 2.6) to perform single crystal X-ray analysis, and the resulting structure confirms the successful synthesis of [Fe_4_Te_4_(SPh)_4_]^3–^. Compound **1** crystallizes in the space group *Fdd*2 (Scheme 1, Table S1†). Since the structural features of the cubic cluster are already thoroughly discussed, we herein refer to the literature.^32^

### NRVS

**Fig. 1:**
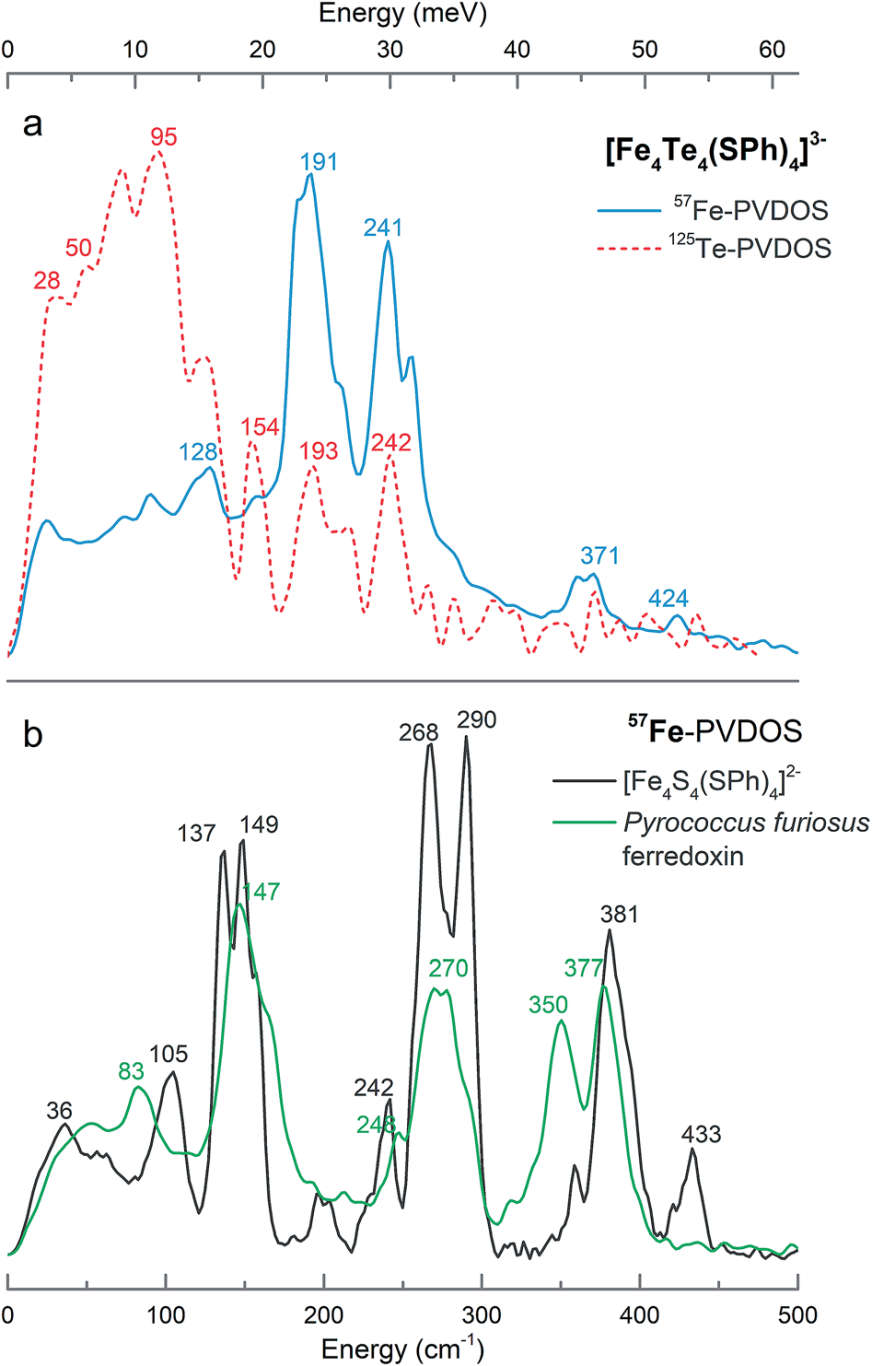
(a) ^125^Te- (red) and ^57^Fe- (blue) PVDOS spectra of (Et_4_N)_3_[^57^Fe_4_^125^Te_4_(SPh)_4_] = **1** in comparison to (b) ^57^Fe-PVDOS profiles of [(*n*-Bu)_4_N]_2_[Fe_4_S_4_(SPh)_4_] (black) and the oxidized D14C mutant of *Pyrococcus furiosus* ferredoxin (green).

Fig. 1a presents the ^125^Te and ^57^Fe partial vibrational density of states (PVDOS) spectra obtained from our NRVS experiments on **1**. The ^57^Fe-PVDOS spectrum shows four main features: a small peak at ∼130 cm^–1^, a global maximum at ∼190 cm^–1^, a doublet at ∼240–250 cm^–1^, and weaker features at ∼360–370 cm^–1^. An even weaker band is resolved at ∼420 cm^–1^. At first glance the ^57^Fe-PVDOS profile of **1** appears to be a completely different spectrum compared to the profiles of [(*n*-Bu)_4_N]_2_[Fe_4_S_4_(SPh)_4_]^34^ and D14C mutant of ferredoxin from *Pyrococcus furiosus* (*Pf* Fd) shown in Fig. 1b.^19^ There seems little resemblance to the ^57^Fe-PVDOS of [4Fe–4S] clusters, which have strong in-core Fe–S stretching bands very approximately in the ∼270–290 and ∼350–380 cm^–1^ regions (Fig. 1b). However, a simple binary harmonic oscillator model for ^57^Fe–^125^Te *vs.*^57^Fe–^32^S vibrations, assuming the same force constant, predicts a frequency downshift factor of 0.72 because of the large ^125^Te mass. This model yields predicted Fe–Te bands in the ∼190–210 cm^–1^ and ∼250–270 cm^–1^ regions, in approximate agreement with the observed ^57^Fe spectrum of **1** (Fig. 1a). Thus, the primary cause of the spectral changes can be attributed to the dramatic change in the chalcogen mass.

The ^125^Te-PVDOS spectrum of **1** reproduces the two main features of the ^57^Fe spectrum at ∼190 cm^–1^ and ∼240 cm^–1^, but at significantly lower intensity. For ^125^Te, the spectrum shows a global maximum at ∼100 cm^–1^ within a broad feature with further local maxima between 20 and 120 cm^–1^. Qualitatively, this indicates relatively little ^125^Te motion in these modes, which are the strongest ^57^Fe features. Even without sophisticated analysis, one can envision the Fe ions moving in a much less mobile matrix of Te ions. In contrast, below 100 cm^–1^ there are modes that show primarily Te motion.

### DFT calculations

#### Structure

**Fig. 2:**
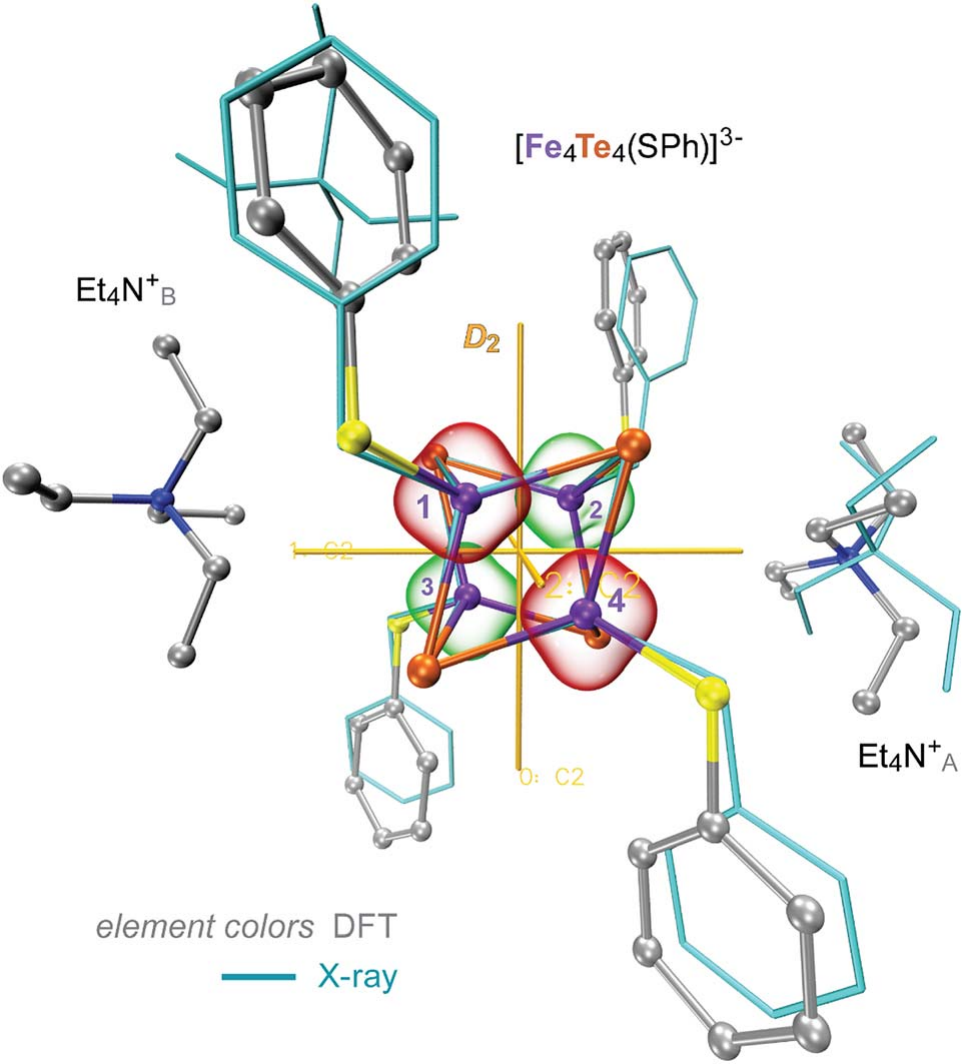
Overlay of the X-ray (wire) and DFT-optimized (ball-and-stick) structures of **1**, combined from the [Fe_4_S_4_(SPh)_4_]^3–^ and 2 × Et_4_N^+^ molecular fragments. 3 × *C*_2_ two-fold axes of the idealized *D*_2_ symmetry of the [4Fe–4Te]^+^ core are shown. Red and green bubbles around the numbered Fe sites correspond to the positive (↑) and negative (↓) 0.01 a.u. point spin density isosurfaces from the representative DFT solution. Hydrogen atoms are omitted for clarity. For extra details, see ESI Fig. S2–S4.†

**Fig. 3:**
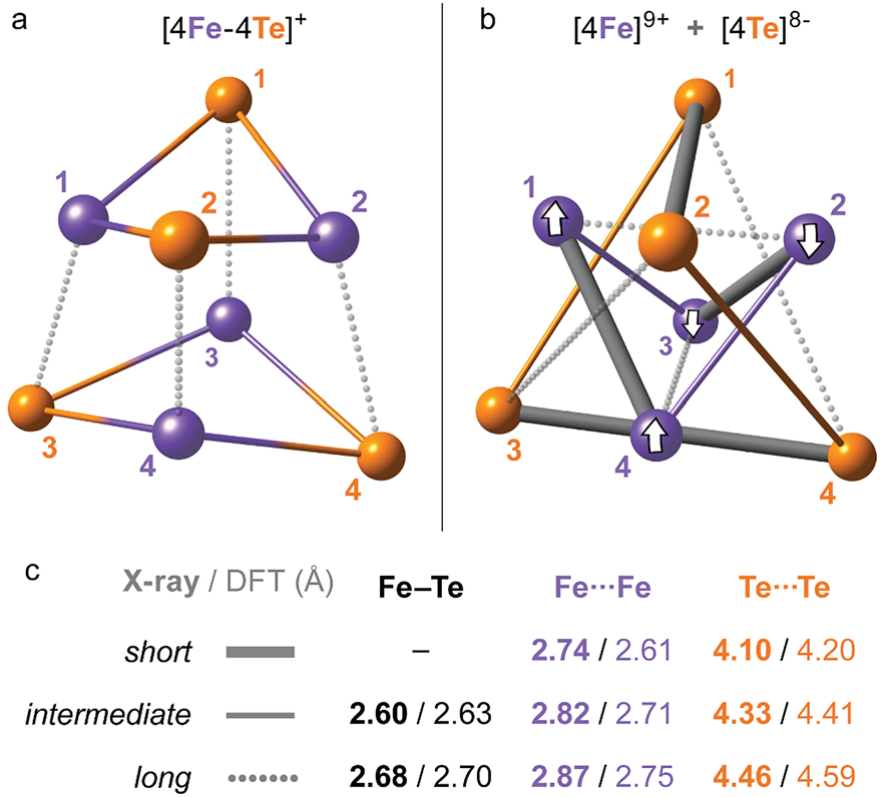
Alternative representations of the iron–tellurium cuboidal core of **1** as (a) an [4Fe–4Te] hexahedron showing 12 × Fe–Te bonding interactions, and (b) a compound of smaller [4Fe] and larger [4Te] tetrahedra showing 6 × Fe···Fe and 6 × Te···Te nonbonding interactions. The internuclear distances are characterized in subsets of ‘long’, ‘intermediate’, and (optionally) ‘short’, with (c) their mean values in the respective polyhedra given as found in the X-ray/DFT-optimized structures. For a detailed account of these distances, see ESI Table S2.† Broken-symmetry (BS) spin alignment (↑ or ↓) of the [4Fe] sites applied in the DFT solution is shown in (b).

DFT modelling has been applied to optimize the structure of **1** (=2 × Et_4_N^+^ + [^57^Fe_4_^125^Te_4_(SPh)_4_]^3–^ here, as explained below) and subsequently generate its normal vibrational modes for NRVS simulation. Based on the present X-ray determination, the model included 2 × Et_4_N^+^ counter ions available from the unit cell as shown in Fig. 2 and S2,† with one of them (Et_4_N_B_^+^) moderately dislocated in the absence of the crystal framework; as detailed later in the text, either inclusion or complete exclusion of counter ions provides qualitatively similar predicted ^125^Te/^57^Fe-PVDOS spectra. The [Fe_4_Te_4_(SPh)_4_]^3–^ molecular fragment is adequately reproduced by the DFT structure, including the positioning of its phenyl rings. The average Fe–Te internuclear distance, the key structural parameter in the [4Fe–4Te]^1+^ core, was reproduced by DFT with a deviation of 0.02 Å only, and the average non-bonding Fe···Fe/Te···Te distances were respectively under-/over-estimated by ∼0.1 Å (Table S2†). Fine structural details of iron-chalcogen cuboids are known to be defined by an interplay of their (i) electronic properties, (ii) ligand identities and their topology, and (iii) effects from the environment, as exemplified by variability in the *S* = 1/2 and 3/2 [Fe_4_X_4_(SR)_4_]^3–^ counterparts of **1**.^32^,^36^–^38^ Earlier characterization of **1** indicated the elongated *D*_2d_ symmetry of its core,^32^ where the elongation is exhibited as 4 ‘long’ Fe–Te distances (Fig. 3a). Indeed, the present X-ray/DFT structures can be fit to idealized *D*_2d_ cuboids with respective RMSD values of 0.06/0.08 Å as shown in Fig. S3a.† However, careful examination of both the X-ray/DFT structures reveals a division of most noticeably the Te···Te and the Fe···Fe distances into three subsets each (Fig. 3b and c), consistent with lowering of the *D*_2d_ symmetry to the *D*_2_ point group, now with even smaller RMSD values of 0.01/0.03 Å as shown in Fig. S3b.† The electronic state of an [4Fe–4X] core is defined by the [4Fe] 3d electronic configuration, where the Fe sites are generally high-spin. Similarly to its iron–sulfur analogue and following the broken-symmetry (BS) formalism,^35^ the configuration in **1** can be described as internally ferromagnetic mixed-valence [2Fe^2.5+^↑] (*S* = 9/2) and ferrous [2Fe^2+^↓] (*S* = 4) pairs, coupled in turn antiferromagnetically (↑↓). We find that localization of the ferromagnetic pairs at sites [Fe_1_, Fe_4_] and [Fe_2_, Fe_3_] (Fig. 2 and 3b) in a BS-DFT solution having total *M*_S_ = 1/2 leads to a description of **1** coherent with the fine distribution of internuclear distances in the X-ray data. Indeed, (i) the spin-dependent delocalization^38^ favoring ferromagnetic coupling is most feasible in the ‘short’ Fe_1_···Fe_4_ and Fe_2_···Fe_3_ interactions as seen in the X-ray structure. Interestingly, the optimized Fe···Fe distances in the [Fe_1_, Fe_4_] and [Fe_2_, Fe_3_] pairs in **1** are essentially invariant with respect to the identity of either the mixed-valence (excess-spin, ↑) or ferrous (↓) pair, unlike *e.g.* the [4Fe–4S]^+^ cluster from the nitrogenase Fe protein developing a ∼0.1 Å shorter contact in its distinct [2Fe^2.5+^↑] pair.^25^ In contrast, (ii) the ‘long’ Fe_1_···Fe_2_ and Fe_3_···Fe_4_ distances are favored by steric repulsion between the in-plane phenyl rings (Fig. 2 and S2†); from DFT, alternative localization of the ferromagnetic Fe sites in the [Fe_1_, Fe_2_] and [Fe_3_, Fe_4_] pairs leads to ∼5 kcal mol^–1^ higher energy and a core structure which does not map well with its X-ray reference. Finally, (iii) the two Et_4_N^+^ counter ions play a role in the *D*_2d_-to-*D*_2_ removal of degeneracy by means of their sandwich-like interaction with the [4Fe–4Te] core; a similar effect can possibly be achieved by specific crystal packing.

In order to compare the [4Fe–4Te]^+^ species to its far more common [4Fe–4S]^+^ counterpart, we likewise analyzed computationally a **1-S** species, where the 4 × Te nuclei of **1** were substituted by 4 × S. This analysis included re-optimization of the **1-S** model, resulting in a more compressed core structure due to the smaller bridging sulfide dimensions (Fig. S4†). In line with earlier X-ray determinations,^32^ the mean DFT-optimized S···S distance in **1-S** is strikingly ∼0.7 Å shorter than the corresponding Te···Te distance in **1**, and the Fe···Fe distances are those least affected (Table S2†). The recognized ‘stereochemical softness’^36^ of the reduced [4Fe–4X]^+^ core is manifested here as a modified distribution of the Fe–X distances when X is either Te (**1**) or S (**1-S**), yet retaining the matching cuboid elongation depicted vertically in Fig. 3a.

#### Spectra

**Fig. 4:**
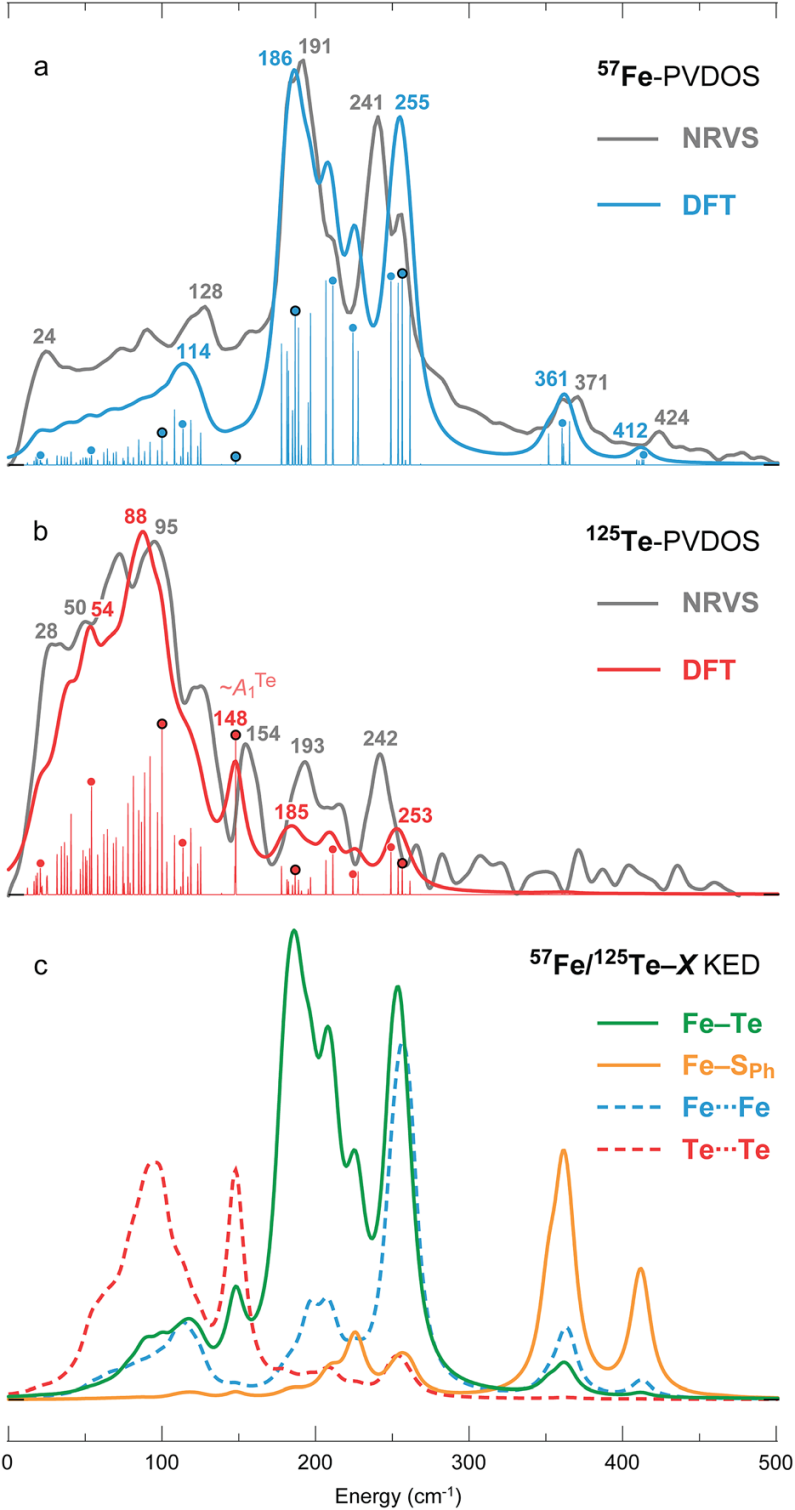
(a) ^57^Fe- and (b) ^125^Te-PVDOS spectra of **1** from NRVS measurements and DFT calculations, followed by (c) DFT-based KED spectra of the relative nuclei motion in bonding 12 × Fe–Te and 4 × Fe–S_Ph_, and non-bonding 6 × Fe···Fe and 6 × Te···Te interactions. Matching NRVS and DFT bands are labelled with their positions. Non-broadened (individual normal mode) DFT PVDOS intensities are additionally provided in a stick-style. For the sticks labelled with dots, corresponding mode animations are available as part of the ESI;† for the four dots circled in black, corresponding modes are depicted in Fig. 5.

The DFT-calculated ^57^Fe- and ^125^Te-PVDOS both follow well the observed NRVS spectra of **1**, having deviations within 15 cm^–1^ between the major observed and calculated band positions as displayed in Fig. 4a and b and S5.† Rigorously unavailable from the experiment, a ^57^Fe/^125^Te ≈ 1.1 ratio between the two PVDOS integral intensities is predicted, implying approximately an equal relative distribution of the [4Fe–4Te]^1+^ vibrational energy between the cationic 4 × Fe and anionic 4 × Te sites. The PVDOS profiles indicate that the vibrational energy of the lighter ^57^Fe nuclei is concentrated mostly within ∼180–260 cm^–1^ (Fig. 4a), and that of the heavier ^125^Te nuclei mostly within ∼50–150 cm^–1^ (Fig. 4b). The two regions are non-overlapping; the coupling between the 4 × Fe and 4 × Te motion is therefore relatively weak. As mentioned in the previous section, NRVS-based ^57^Fe/^125^Te-PVDOS nevertheless display complementary bands implying correlated Fe and Te vibrations at, respectively, 191/193 and 241/242 cm^–1^; the DFT-based ^57^Fe/^125^Te-PVDOS reproduces these features at 186/185 and 255/253 cm^–1^ (Fig. 4a and b).

A smaller DFT model **1′** = [Fe_4_Te_4_(SPh)_4_]^3–^, which lacks the Et_4_N^+^ counter ions, has been considered additionally. Both the more complete **1** and the size-reduced **1′** models produce qualitatively similar PVDOS spectra as shown in Fig. S5.† Inclusion of the counter ions in **1** (*vs.* exclusion in **1′**), however, provides a more realistic distribution of the intensities in the 180–260 cm^–1^ range of the ^57^Fe-PVDOS, and as well a better population of the low-frequency <100 cm^–1^ portion of the ^125^Te-PVDOS.

DFT-based kinetic energy distribution (KED) spectra shown in Fig. 4c facilitate analysis of the normal modes in terms of relative displacements in the nuclei pairs. The calculated ^57^Fe-PVDOS spectrum is largely reproduced by the Fe–Te KED profile, implying that the Fe nuclei vibrate in a predominantly static framework of the Te nuclei. The ^125^Te-PVDOS is in contrast similar to the ‘Te-only’ Te···Te profile, as if the Te nuclei moved independently. ^57^Fe nuclei vibrations in the stronger Fe–S_Ph_ (*vs.* weaker Fe–Te) bonds produce the two NRVS/DFT high-end bands at 371/361 and 424/412 cm^–1^, respectively (Fig. 4a).

**Fig. 5:**
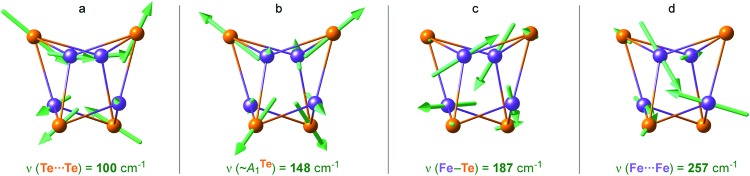
Arrow-style representations of selected calculated normal modes in the [4Fe–4Te]^+^ core of **1**, having (a) Te···Te, (b) ‘breathing’ ∼*A*_1_^Te^, (c) Fe–Te, and (d) Fe···Fe character. The arrow sizes are proportional to the nuclei displacement amplitudes; actual amplitudes in (b) are respectively ∼0.02/0.005 Å for Te/Fe. Animations for these and several other normal modes are provided as part of the ESI.†

Breakdown of the calculated PVDOS into contributions from individual normal modes, see Fig. 4a and b and 5, provide further characterization of **1**. Of remarkable note is a mode calculated at 148 cm^–1^ and having prominent ^125^Te-PVDOS (and Te···Te KED) intensity, yet vanishingly weak in ^57^Fe-PVDOS. Centered in the broad ∼50 cm^–1^ region devoid of other bands, the 148 cm^–1^ mode directly corresponds to the experimental ^125^Te band observed at 154 cm^–1^. DFT reveals this as a pseudo-*A*_1_^Te^ (totally-symmetric) character ‘breathing’ of the [4Te] tetrahedron depicted in Fig. 5b, a type of vibration well-known from studies on [4Fe–4S] and other cubic clusters.^19^,^34^,^39^

**Fig. 6:**
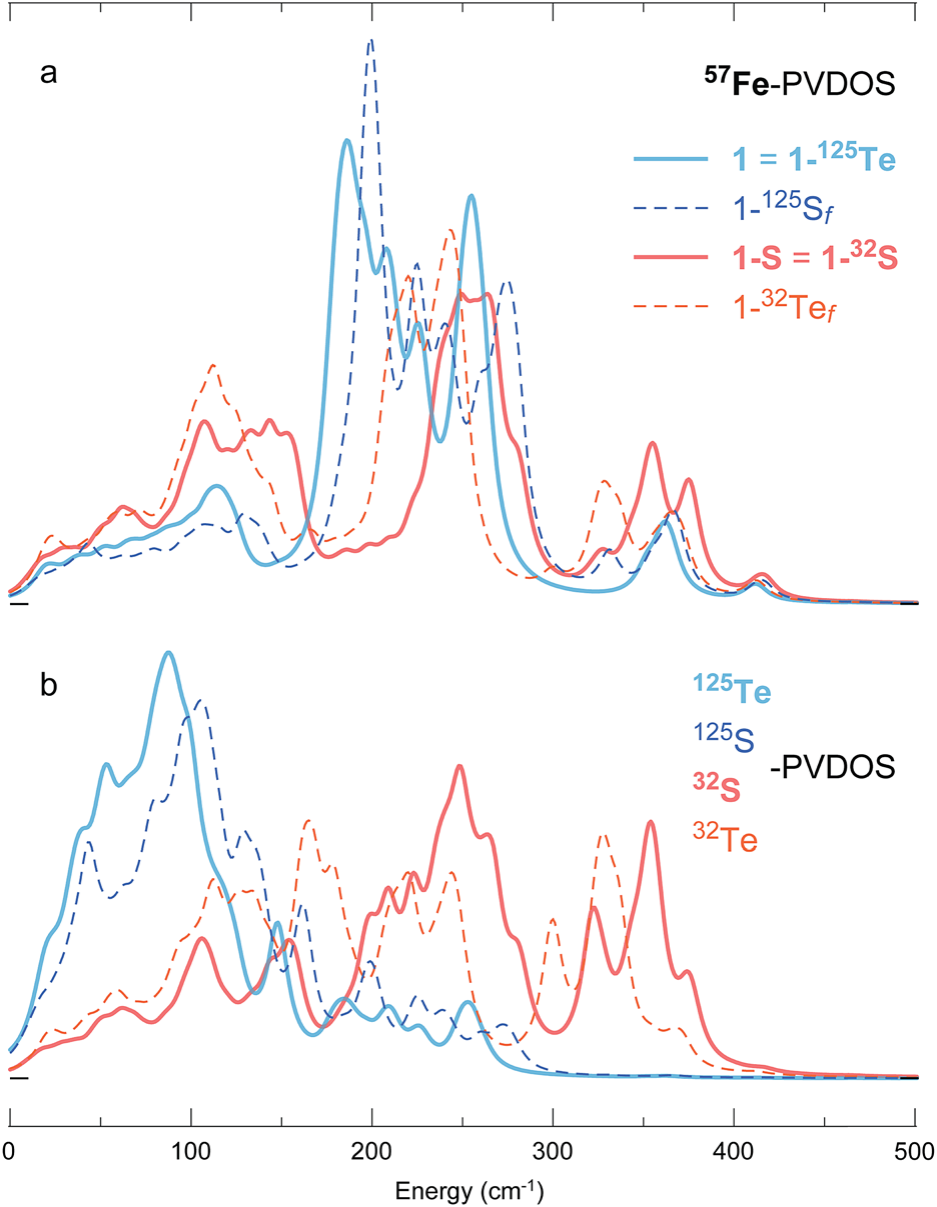
DFT-simulated (a) ^57^Fe- and (b) ^125/32^Te/S-PVDOS spectra from models **1** (=**1-^125^Te**) and **1-S** (=**1-^32^S**), as well as their fictitious ^32^Te- and ^125^S-isotopologues **1-^32^Te*_f_*** and **1-^125^S*_f_***.

To disentangle the (i) structural/electronic properties and (ii) nuclear masses in the effects determining the PVDOS, we introduce two additional DFT systems **1-^32^Te*_f_*** and **1-^125^S*_f_*** having *fictitious* isotope nuclei with the ‘native’ Te and S masses interchanged, yet their structures correspondingly equal to **1** = **1-^125^Te** and **1-S** = **1-^32^S**. As seen from the PVDOS comparison in Fig. 6, the **1-^125^Te** and **1-^125^S*_f_*** systems, despite their elementary (and thus chemically) different content and different structures, display very similar pairs of both ^57^Fe- and ^125^Te(^125^S)-PVDOS profiles; a blue-shift of ∼15–20 cm^–1^ in the **1-^125^S*_f_****vs.***1-^125^Te** bands is explained by the shorter Fe–S *vs.* Fe–Te distances (Fig. S4 and Table S2†). In contrast, the variation is more prominent between the **1-^32^S** and **1-^32^Te*_f_*** DFT spectra, particularly in the ^32^S(^32^Te)-PVDOS. The ^57^Fe-PVDOS intensities in the **1-^32^S**(**^32^Te*_f_***) systems become more evenly distributed over the entire spectral range *vs.* sharper bands of **1-^125^Te**(**^125^S*_f_***) consolidated around 180–280 cm^–1^, as seen in Fig. 6a. Displaying similarities to the [4Fe–4S] NRVS spectra observed earlier,^19^,^34^ see spectra in Fig. 1b, this behaviour of **1-^32^S**(**^32^Te*_f_***) is explained by a stronger vibrational coupling between the motion of Fe and the lighter 32-mass (*vs.* heavier 125-mass) nuclei in the cubic core. The naturally relevant **1-^32^S** system shows the strongest coupling, producing ^57^Fe- (Fig. 6a) and ^32^S-PVDOS (Fig. 6b) profiles that nearly follow each other.

#### Redox-dependent properties

The synthetic [4Fe–4S] phenylthiolate-coordinated analogue to the [4Fe–4Te] species **1** has been characterized by ^57^Fe-NRVS earlier, as shown in Fig. 1b,^34^ albeit in the oxidized [4Fe–4S]^2+^ state (in contrast to the above described **1-S** model that has the reduced [4Fe–4S]^1+^ core); adding credibility to the present DFT methodology, the calculated spectrum of the oxidized model [**1-S**]^+^ compares well to the experimental line shape as shown in Fig. S6a.†

The ^57^Fe-PVDOS comparison drawn above for the reduced **1***vs.***1-S** DFT models (Fig. 6a) applies as well to their 1e^–^ oxidized [**1**]^+^*vs.* [**1-S**]^+^ counterparts (Fig. S6a *vs.* b†). Both Te/S chalcogenide alternatives display only minor (Te-variant) to moderate (S-variant) redox-dependent alterations in their spectra, where the S-variant behaves similar to the [4Fe–4S]^+/2+^ cofactor from ferredoxin characterized by ^57^Fe-NRVS earlier.^19^,^20^ The major bands display blue shifts within 10 cm^–1^ upon oxidation, explained by +0.8/+1.5% larger volume of respectively the [4Fe–4Te/S]^1+^*vs.* [4Fe–4Te/S]^2+^ cores (Table S3†). While these redox-dependent changes can be considered as insignificant, relative trends in the ^57^Fe-PVDOS and core volume adjustments indicate that the S-to-Te substitution may present a cubic cluster having enhanced proficiency in electron transfer. This expectation is now supported by a significant ∼1.5 ratio (S/Te-variant) in the calculated reorganization energies of structural relaxation following electron transfer for the **1-S**/[**1-S**]^+^ (17.4 kJ mol^–1^) *vs.***1**/[**1**]^+^ (11.6 kJ mol^–1^) redox couples (Table S4†). The [4Fe–4Te] core with its volume ∼1.4 times larger than that of [4Fe–4S] (Table S3†) is therefore expected to be more efficient in charge delocalization.

## Conclusions

Iron–sulfur clusters play many important roles in biological systems, ranging from purely structural functions to electron transfer, small molecule sensing, and as components of active sites. However, the large amount of sulfur in most biological samples poses a challenge for spectroscopists who want to investigate these clusters from the sulfur point of view.

We have shown here that sulfur to tellurium exchange, combined with NRVS measurements from both ^57^Fe and ^125^Te points of view, creates a powerful tool for chalcogenide-specific vibrational spectroscopy. Additionally, DFT calculations reproduced and rationalized the obtained structure and spectra. Our results clearly demonstrate corresponding bands in ^57^Fe- and ^125^Te-NRVS spectra that could successfully be assigned to the same normal modes. Unlike [4Fe–4S] clusters where the vibrational coupling of the Fe and S motion is tight, a significant difference in the Fe *vs.* Te nuclei masses and slightly weaker Fe–Te force constants in the [4Fe–4Te] cuboid both lead to divergence in the vibrational signatures of the [4Fe] and [4Te] interposed tetrahedra. Herein, this exchange leads to clearly isolated Fe-only motions displaying sharper bands within the ^57^Fe spectrum by almost cancelling out the Fe–Te vibrational coupling. Thus, (selective) Te incorporation can be potentially used to simplify complex ^57^Fe-NRVS spectra of proteins containing multiple Fe–S clusters, and to allow for a more discrete discussion on the properties of such active centers.

This work is the first ^125^Te-NRVS investigation of an isolated [4Fe–4Te] cluster, and it lays the basis for bioinorganic spectroscopic investigation of Te substitution. Our disclosure of the [4Te]-‘breathing’ mode in **1** is an example of finding by ^125^Te-NRVS which would evade determination by ^57^Fe-NRVS which is commonly used nowadays. Se substitution for S in biological [2Fe–2S]^40^ and [4Fe–4S]^41^ clusters have been known for decades,^42^ and Te substitution in various electron-transport proteins as well as enzyme active sites should be feasible. For example, a [4Fe–4Se] cluster was shown to replace the [4Fe–4S]_H_ sub-cluster in [FeFe]-hydrogenase with full retention of activity.^43^ [4Fe–4Te] clusters could presumably be incorporated into hydrogenase in the same manner and studied by ^125^Te-NRVS. Since Se can also be incorporated into thiolate positions of the H-cluster azadithiolate ligand,^44^ comparable experiments using Te and ^125^Te-NRVS can also be envisaged. In small molecules, Te can be a replacement for terminal thiolate ligands or thioethers, although it remains to be seen whether tellurocysteine or telluromethionine can be inserted into proteins in the manner already established for selenocysteine. Finally, Se has been shown to incorporate into the nitrogenase MoFe cofactor,^45^ and the analogous experiments with Te might be interesting. Although it has a larger ionic radius and lower electronegativity, tellurium often exhibits chemistry similar to sulfur, and it can replace sulfur in many structures. Our calculations for the [4Fe–4Te] *vs.* [4Fe–4S] clusters indicate that, while iron–tellurium cofactors are bigger in their size, they have lower reorganization energies favorable during events of electron transfer. These results clearly showcase the potential of ^125^Te-NRVS in such modified systems.

## Conflicts of interest

There are no conflicts of interest to declare.

## Supplementary Material

Supplementary information

Supplementary information

Supplementary information

Crystal structure data
