# Supplementary material for: Insights from 125Te and 57Fe nuclear resonance vibrational spectroscopy: a [4Fe–4Te] cluster from two points of view
Source: Chem Sci. 2019 Jun 24;10(32):7535–41. doi: 10.1039/c9sc02025j (PMC6761874; doi:10.1039/c9sc02025j)
Supplement: Supplementary file 1 [file SC-010-C9SC02025J-s001.pdf]

Electronic Supplementary Material (ESI) for Chemical Science.
This journal is © The Royal Society of Chemistry 2019

Insights from ^{125}Te and ^{57}Fe Nuclear Resonance Vibrational Spectroscopy: a [4Fe-4Te] Cluster from Two Points of View

Florian Wittkamp,[† a] Nakul Mishra,[† b] Hongxin Wang,[b] Hans-Christian Wille,[c] René Steinbrügge,[c] Martin Kaupp,[d] Stephen P. Cramer,*[b] Ulf-Peter Apfel,*[a,e] and Vladimir Pelmenschikov*[d]

[a] Department of Chemistry and Biochemistry, Inorganic Chemistry I, Ruhr-Universität Bochum, Universitätsstraße 150, 44801 Bochum, Germany

[b] Department of Chemistry, University of California, Davis, One Shields Avenue, Davis, California 95616, United States

[c] Deutsches Elektronen-Synchrotron DESY, Notkestraße 85, 22607 Hamburg, Germany

[d] Institute of Chemistry, Theoretical Chemistry/Quantum Chemistry, Technical University of Berlin, Sekr. C7, Straße des 17. Juni 135, 10623 Berlin, Germany

[e] Fraunhofer UMSICHT, Osterfelder Straße 3, 46047 Oberhausen, Germany

Supporting Information

Supplementary Tables

Table S1. Crystal data and structure refinement for **1**. S2

Table S2. Important internuclear distances in **1**(-**S**) from X-ray spectroscopy and DFT calculations. S3

Table S3. Total volumes and their cross-ratios for DFT models **1**, [**1**]$^+$, **1-S**, and [**1-S**]$^+$ and their [4Fe-4Te/S]$^{+/2+}$ cores. S4

Table S4. Reorganization energies calculated for redox couples **1**/[**1**]$^+$ and **1-S**/[**1-S**]$^+$. S4

Supplementary Figures

Figure S1. ^{1}H-NMR Spectrum of **1.** S5

Figure S2. Overlay of the X-ray and DFT-optimized structures of **1**. S6

Figure S3. DFT-optimized structure of **1** fit to the D_{2d} and D_2 point group symmetries. S7

Figure S4. Magnified overlay of the [4Fe-4Te(S)]$^+$ core fragments of **1**(-**S**) from the X-ray data and DFT optimization. S8

Figure S5. ^{57}Fe- and ^{125}Te-PVDOS spectra of **1** and **1'**. S9

Figure S6. ^{57}Fe-PVDOS spectra of (a) **1-S** and [**1-S**]$^+$ vs (b) **1**(-**Te**) and [**1-Te**]$^+$. S10

Materials and Methods

X-ray Data Collection and Structure Solution Refinement S11

General Synthetic Methods S11

Syntheses S11

Nuclear Resonance Vibrational Spectroscopy S11

DFT Methods S12

References

S12

Table S1. Crystal data and structure refinement for **1**.

Identification code	$(NEt_4)_3[Fe_4Te_4(SPh)_4]$ = 1
Empirical formula	$C_{38.4}H_{64}Fe_{3.2}N_{2.4}S_{3.2}Te_{3.2}$
Formula weight	1248.95
Temperature/K	170(10)
Crystal system	orthorhombic
Space group	Fdd2
a/Å	25.0146(9)
b/Å	39.6995(13)
c/Å	12.1800(4)
α/°	90
β/°	90
γ/°	90
Volume/Å^3	12095.6(7)
Z	10
ρ_{calc}g/cm^3	1.715
μ/mm^{-1}	3.002
F(000)	6120.0
Crystal size/mm^3	0.3 × 0.3 × 0.3
Radiation	MoKα (λ = 0.71073)
2Θ range for data collection/°	6.01 to 54.998
Index ranges	$-32 \le h \le 32$, $-51 \le k \le 51$, $-15 \le l \le 15$
Reflections collected	74687
Independent reflections	6945 [R_{int} = 0.0556, R_{sigma} = 0.0333]
Data/restraints/parameters	6945/1/291
Goodness-of-fit on F^2	1.089
Final R indexes [I>=2σ (I)]	R_1 = 0.0259, wR_2 = 0.0522
Final R indexes [all data]	R_1 = 0.0313, wR_2 = 0.0548
Largest diff. peak/hole / e Å^{-3}	0.76/-0.38
Flack parameter	-0.034(11)
CCDC Deposit	1908709

Table S2. Important bonding (–) and non-bonding (···) internuclear distances and their mean values (Å) from X-ray spectroscopy and DFT calculations characterizing the 4×S_{Ph}-coordinated $[4Fe\text{-}4Te]^{+}$ and $[4Fe\text{-}4S]^{+}$ core structures of species $(Et_4N)_3[Fe_4Te_4(SPh)_4]$ = **1** and $(Et_4N)_3[Fe_4S_4(SPh)_4]$ = **1-S** respectively. The detailed X-ray data is tabulated only for the present determination of species **1**; mean distances from independent X-ray studies on species closely related to **1** and **1-S** are given inside the ' [ ] ' square brackets. Distribution of the distances into '*short*' (**bold** type), '*intermediate*' (normal type), and '*long*' (gray typeface) sets within each group is indicated, where the intermediate ones are closest to the total mean value in the respective group. In the mean distances rows, ' × ' numbers in each set apply to the present X-ray and DFT structures of **1** only.

i‡	*j*‡	1 X-ray	1 DFT	1-S X-ray&	1-S DFT
	**Fe$_i$ – Te(S)$_j$ ***				
1$ –	1	2.60	2.61		**2.26#**
	2	2.60	2.64		2.35
	3	2.69	2.69		2.37
2 –	1	2.62	2.65		2.32
	2	2.59	2.64		**2.30#**
	4	2.67	2.72		2.37
3 –	1	2.69	2.69		2.36
	3	2.60	2.64		**2.30#**
	4	2.60	2.63		2.32
4$ –	2	2.67	2.70		2.37
	3	2.62	2.64		2.34
	4	2.59	2.61		**2.26#**
Mean	***– short***	–	–		***2.28***
	×8 intermediate	*2.60*	*2.63*		*2.33*
	×4 long	*2.68*	*2.70*		*2.37*
	total	*2.63 [2.63]&*	*2.65*	*[2.31]&*	*2.33*
	**Fe$_i$ ··· Fe$_j$**				
1$ ···	2	2.87	2.75		2.69
	3	2.83	2.74		2.70
	4$	**2.74**	**2.59**		**2.61#**
2 ···	3	**2.74**	**2.62**		**2.64**
	4$	2.80	2.69		2.70
3 ···	4$	2.87	2.76		2.68
Mean	***×2 short***	***2.74***	***2.61***		***2.62***
	×2 intermediate	*2.82*	*2.71*		*2.69*
	×2 long	*2.87*	*2.75*		–
	total	*2.81 [2.82]&*	*2.69*	*[2.74]&*	*2.67*
	**Te(S)$_i$ ··· Te(S)$_j$ ***				
1 ···	2	**4.10**	**4.21**		**3.65**
	3	4.34	4.38		3.68
	4	4.46	4.58		3.85
2 ···	3	4.46	4.59		3.89
	4	4.33	4.45		3.68
3 ···	4	**4.10**	**4.19**		**3.64**
Mean	***×2 short***	***4.10***	***4.20***		***3.64***
	×2 intermediate	***4.33***	*4.41*		*3.68*
	×2 long	*4.46*	*4.59*		*3.87*
	total	*4.30 [4.3]&*	*4.40*	*[3.66]&*	*3.73*
	**Fe$_i$ – S$_{Phj}$**				
1$ –	1	2.30	2.34		2.33
2 –	2	2.31	2.30		2.31
3 –	3	2.30	2.32		2.31
4$ –	4	2.31	2.33		2.32
Mean	*total*	*2.30 [2.29]&*	*2.32*	*[2.30]&*	*2.32*

‡ Atomic Fe/Te(S) numbering of the cuboid core follows Fig. S2 (and other figures); the S_{Ph} ligands are numbered following their coordinated Fe sites.

***** Te(S) implies Te for **1** and S for **1-S**.

$ Fe_1 and Fe_4 sites that are spin-up in the representative state used in the DFT calculations.

\# Fe–S and Fe–Fe distances categorized earlier[1] as ***short*** in the cysteine-coordinated $[4Fe\text{-}4S]^{1+}$ cluster of nitrogenase Fe protein, provided the matching Fe sites spin alignment.

& Mean distances from independent X-ray determinations of **1**, and variant of **1-S** having Et_3MeN^{+} counter ions instead of Et_4N^{+}.[2, 3]

Table S3. Total volumes (Å^3) and their cross-ratios (" **/** ") for DFT models **1**, [**1**]$^+$, **1-S**, and [**1-S**]$^+$ (**gray** typeface), as well as their [4Fe-4Te/S]$^{+/2+}$ iron-chalcogen cores (normal typeface), organized in a tabular format. The values are based on Van der Waals cavities generated during SCRF calculations as described in the Materials and Methods section.

**1** = 1303 Å^3<br>[4Fe-4Te]$^+$ = 253 Å^3	[**1**]$^+$ = 1310 Å^3<br>[4Fe-4Te]$^{2+}$ = 251 Å^3	**1 /** [**1**]$^+$ = 0.995<br>[4Fe-4Te]$^+$ **/** [4Fe-4Te]$^{2+}$ = 1.008
**1-S** = 1236 Å^3<br>[4Fe-4S]$^+$ = 186 Å^3	[**1-S**]$^+$ = 1251 Å^3<br>[4Fe-4S]$^{2+}$= 183 Å^3	**1-S /** [**1-S**]$^+$ = 0.988<br>[4Fe-4S]$^+$ **/** [4Fe-4S]$^{2+}$ = 1.015
**1 / 1-S** = 1.055<br>[4Fe-4Te]$^+$ **/** [4Fe-4S]$^+$ = 1.360‡	[**1**]$^+$ **/** [**1-S**]$^+$ = 1.047<br>[4Fe-4Te]$^{2+}$ **/** [4Fe-4S]$^{2+}$ = 1.370	

‡ As compared to = 1.47 (DFT) or = 1.48 (X-ray) volume ratios based on approximating the mean Fe–Te/S bonding distances (Table S2) to edges of idealized cubes.

Table S4. Reorganization energies calculated for redox couples **1**/[**1**]$^+$ and **1-S**/[**1-S**]$^+$ (respectively corresponding to the [4Fe-4Te]$^{+/2+}$ and [4Fe-4S]$^{+/2+}$ cubic cores) during oxidation (λ_{ox}), reduction (λ_{red}), and the overall redox process ($\lambda = \lambda_{red} + \lambda_{ox}$) following the protocol originally by Ryde et al.[4, 5] Notably, *absolute* theoretical reorganization energies may vary significantly depending on the method, model setup, and environment treatment;[6-8] these are the *relative λ* values for the Te- vs S-variant species that play importance in the present work, based on the uniform DFT setup as described in the Materials and Methods section.

	Reorganization Energy (kJ mol^{-1})				
	$\boldsymbol{\lambda_{red}}$‡	+	$\boldsymbol{\lambda_{ox}}$*	=	$\boldsymbol{\lambda}$
**1**/[**1**]$^+$ ([4Fe-4Te]$^{+/2+}$ core)	5.9		5.7		11.6
**1-S**/[**1-S**]$^+$ ([4Fe-4S]$^{+/2+}$ core)	8.8		8.7		17.4$

‡ Reorganization energy during reduction is the difference between the energies of the reduced complex in its equilibrium geometry (Red^{Red}) and reduced complex at the equilibrium structure of the oxidized complex (Ox^{Red}), $\lambda_{red} = E(Red^{Red}) - E(Ox^{Red})$.

* Reorganization energy during oxidation is the difference between the energies of the oxidized complex in its equilibrium geometry (Ox^{Ox}) and oxidized complex at the equilibrium structure of the reduced complex (Red^{Ox}), $\lambda_{ox} = E(Ox^{Ox}) - E(Red^{Ox})$.

$ As compared to λ = 18.8 kJ mol^{-1} for the [4Fe-4S]$^{+/2+}$(Cys$_4$) cluster from D14C mutant of *Pyrococcus furiosus* ferredoxin calculated using very similar methodology.[6]

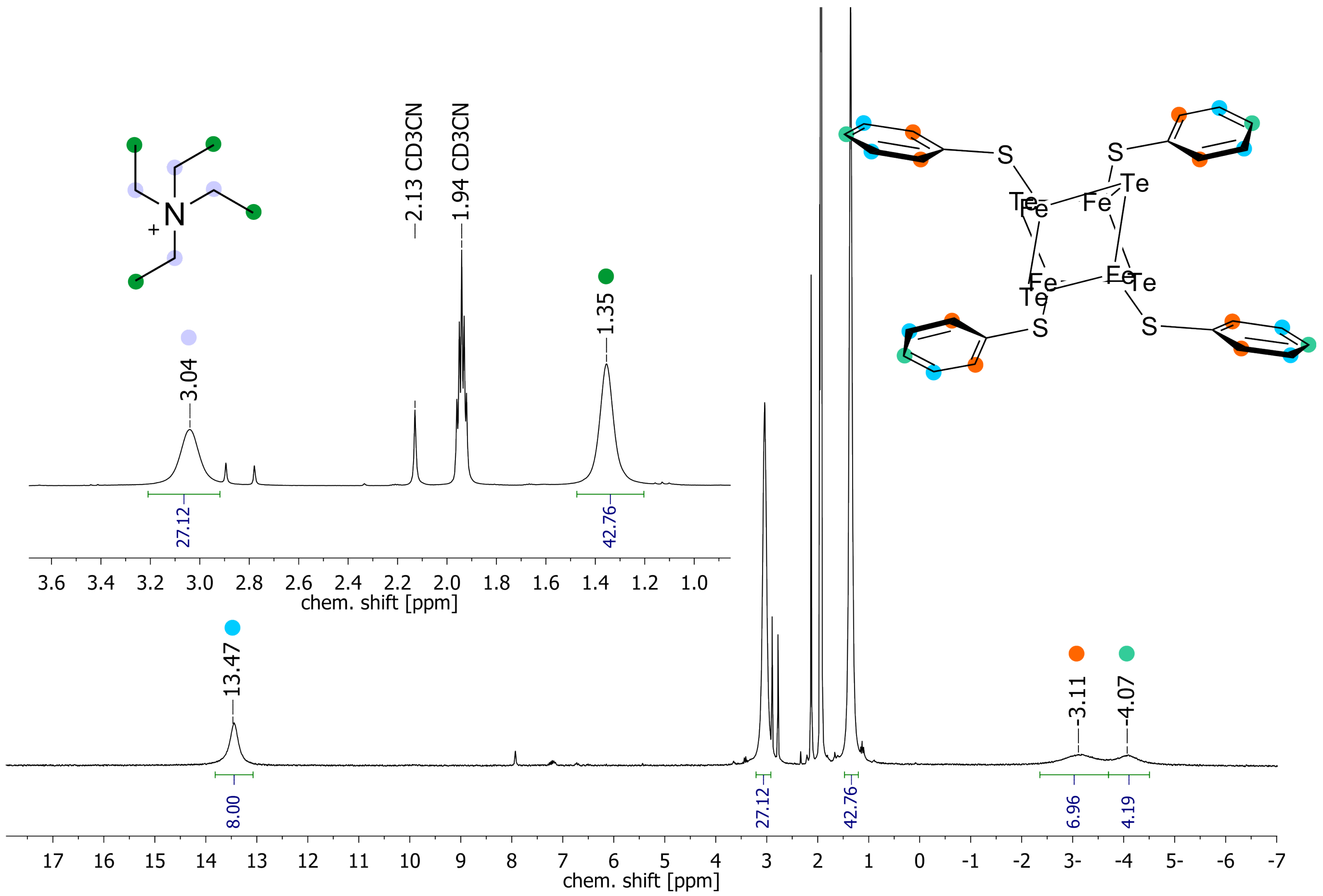

Figure S1. ^{1}H-NMR Spectrum of **1**. Associations between proton signals (in the spectra) and carbon sites binding these protons (in the structure) are shown using color codes.

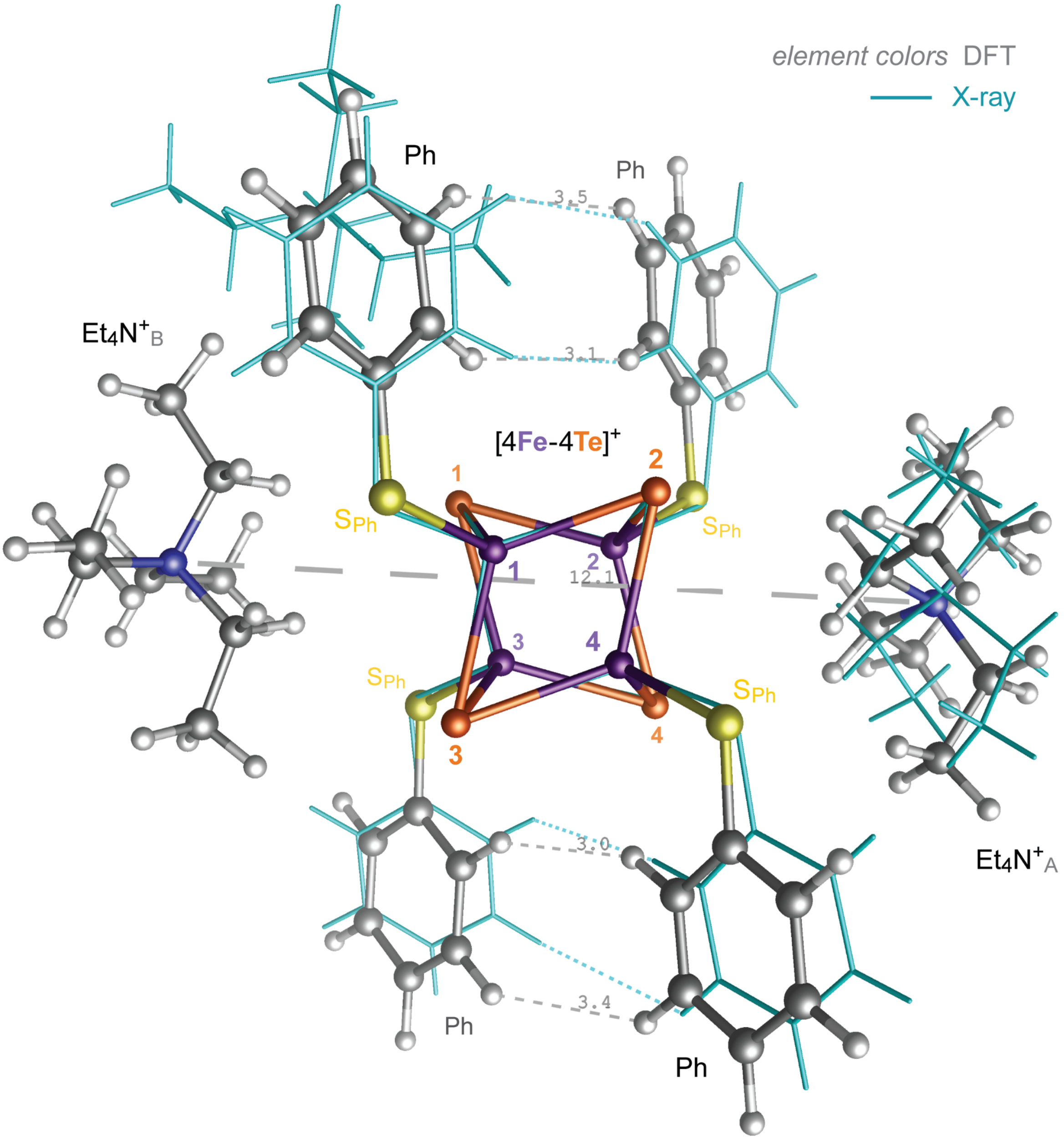

Figure S2. Overlay of the X-ray (blue, wire) and DFT-optimized (element colors, ball-and-stick) structures of **1**, combined from the $[Fe_4S_4(SPh)_4]^{3-}$ and $2{\times}Et_4N^+$ molecular fragments. The two structures were superimposed using positions of the $4{\times}S_{Ph}$ ligand nuclei. The system topology is clarified using interatomic distance (Å) measurements (gray, DFT-optimized data) between protons of the neighboring phenyl rings, and nitrogen atoms in the counter ions. This figure complements Fig. 2 of the main text.

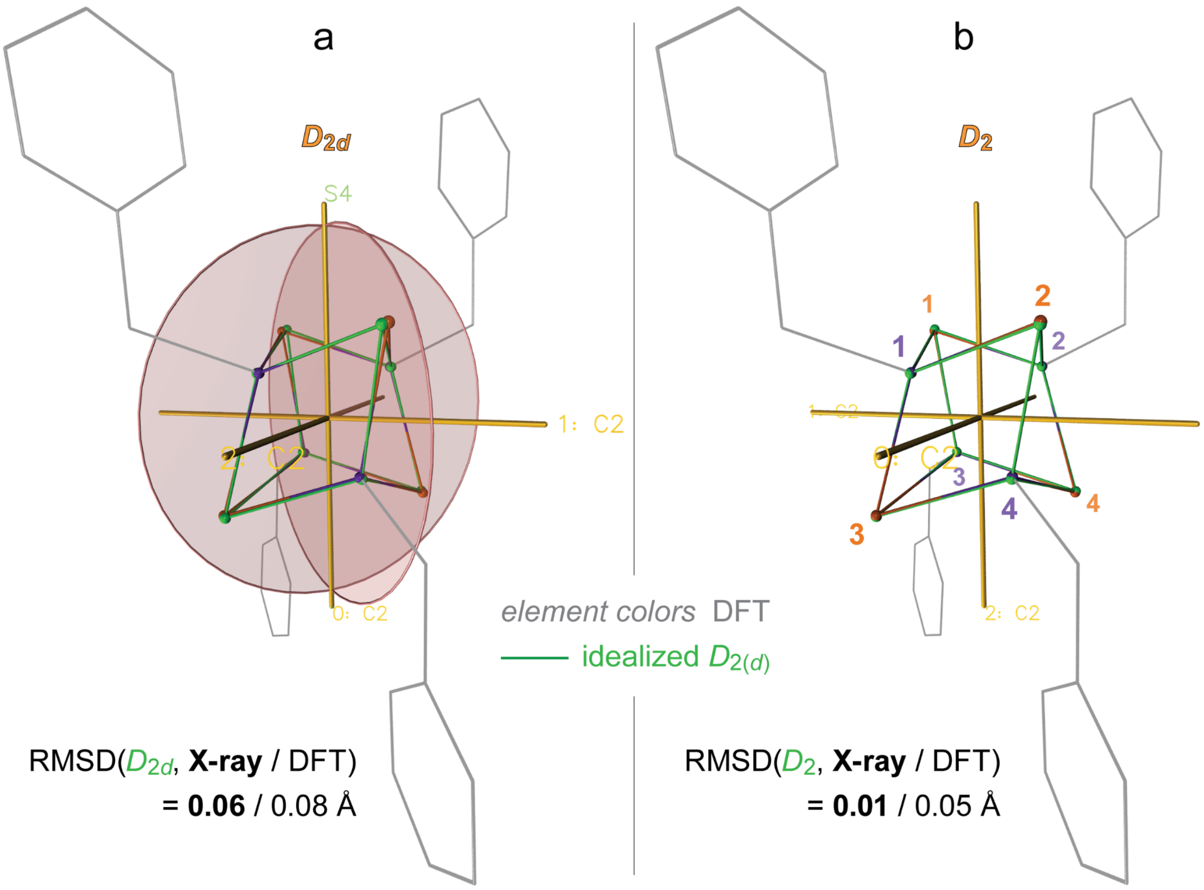

Figure S3. DFT-optimized structure of **1** fit to the (a) D_{2d} and (b) D_2 point group symmetries using the positions of the [4Fe-4Te] core nuclei. The counter ions and hydrogen atoms are omitted for clarity. The symmetry elements shown are (a, b) 3×C_2 two-fold axes, (a) 2×σ_d diagonal mirror planes (approximately coinciding with the Ph ring pairs), and (a) S_4 four-fold anti- and clockwise rotoreflection axis. The idealized $D_{2(d)}$ core structures are shown in green. Similar analysis applies as well to the presently obtained X-ray structure. The RMSD values (Å) between the X-ray / DFT-optimized [4Fe-4Te] structures and their idealized counterparts are given.

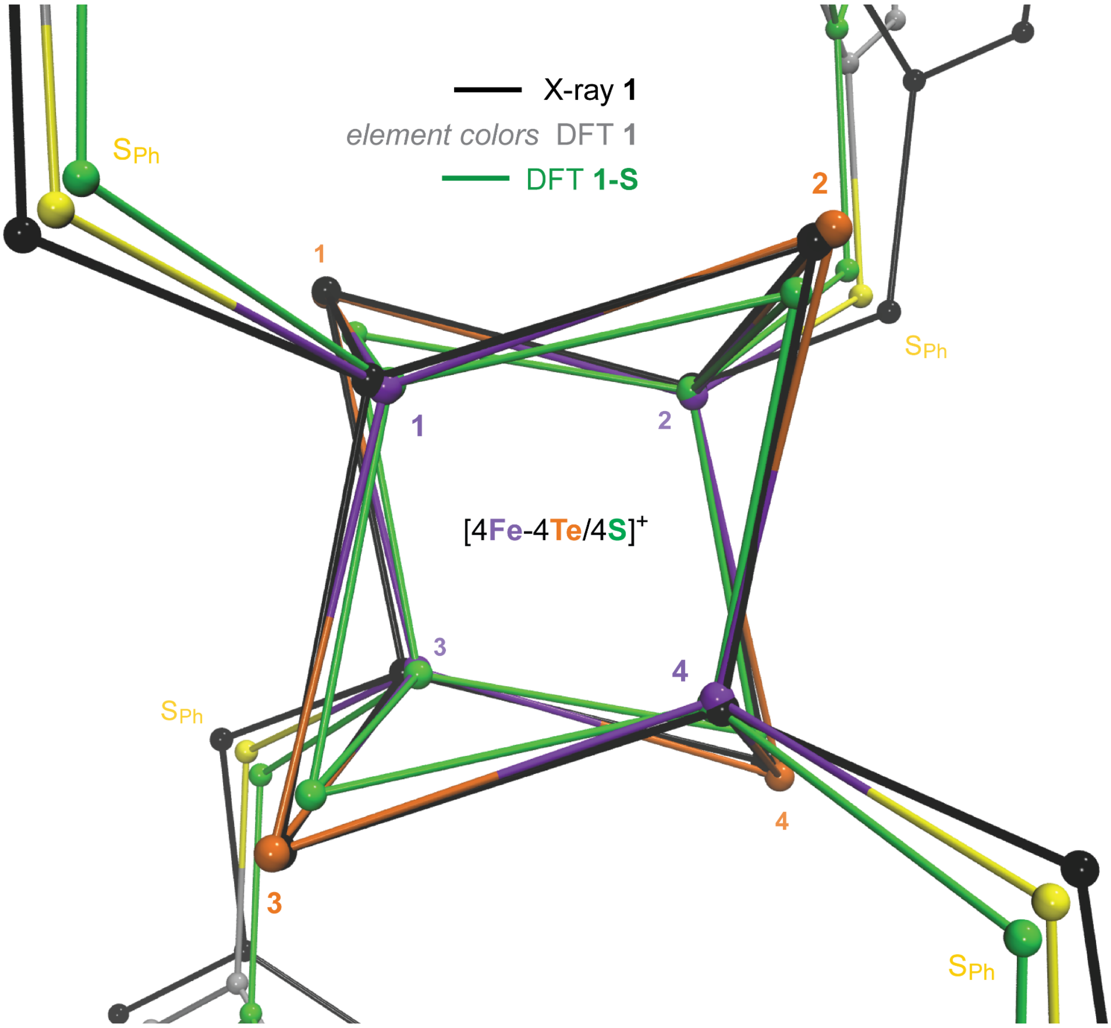

Figure S4. Magnified overlay of the [4Fe-4*X*]$^+$ core fragments of **1** (*X* = Te) from the X-ray data (black) and DFT optimization (element colors), and that of **1-S** (*X* = S) from the DFT optimization (green). The three structures were superimposed using positions of the 4×S_{Ph} ligand nuclei.

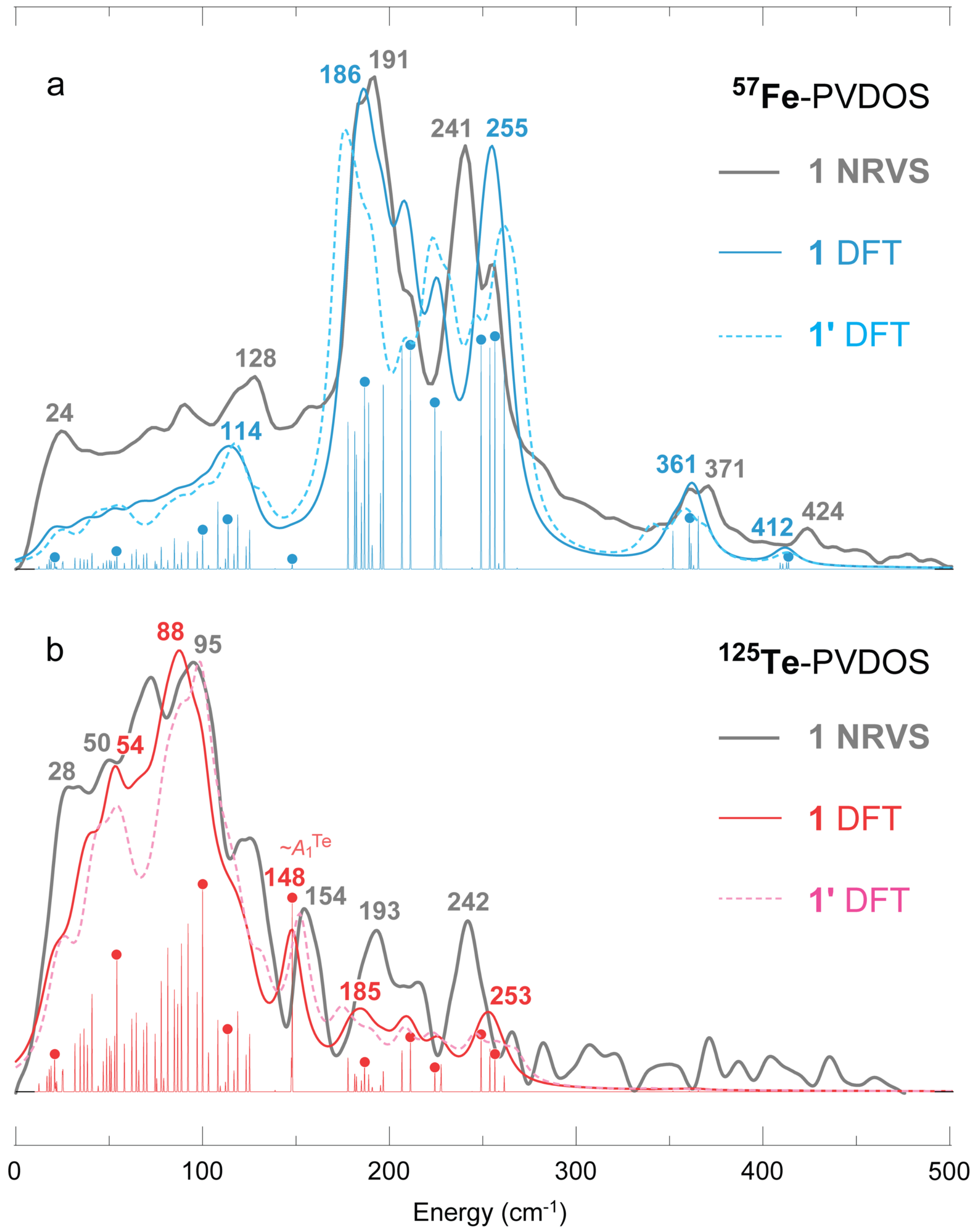

Figure S5. (a) ^{57}Fe- and (b) ^{125}Te-PVDOS spectra of **1** from NRVS measurements and DFT calculations. Tentatively matching NRVS and DFT bands are labelled with their positions. Non-broadened (individual normal mode) DFT intensities are additionally provided in stick-style. For the modes labelled with dots, animations are available as part of the ESI†. DFT spectra of model **1'** = $[Fe_4Te_4(SPh)_4]^{3-}$ which lacks the $N(Et)_4^+$ tetraethylammonium counter ions is additionally provided in broken lines. This figure complements Fig. 4 of the main text.

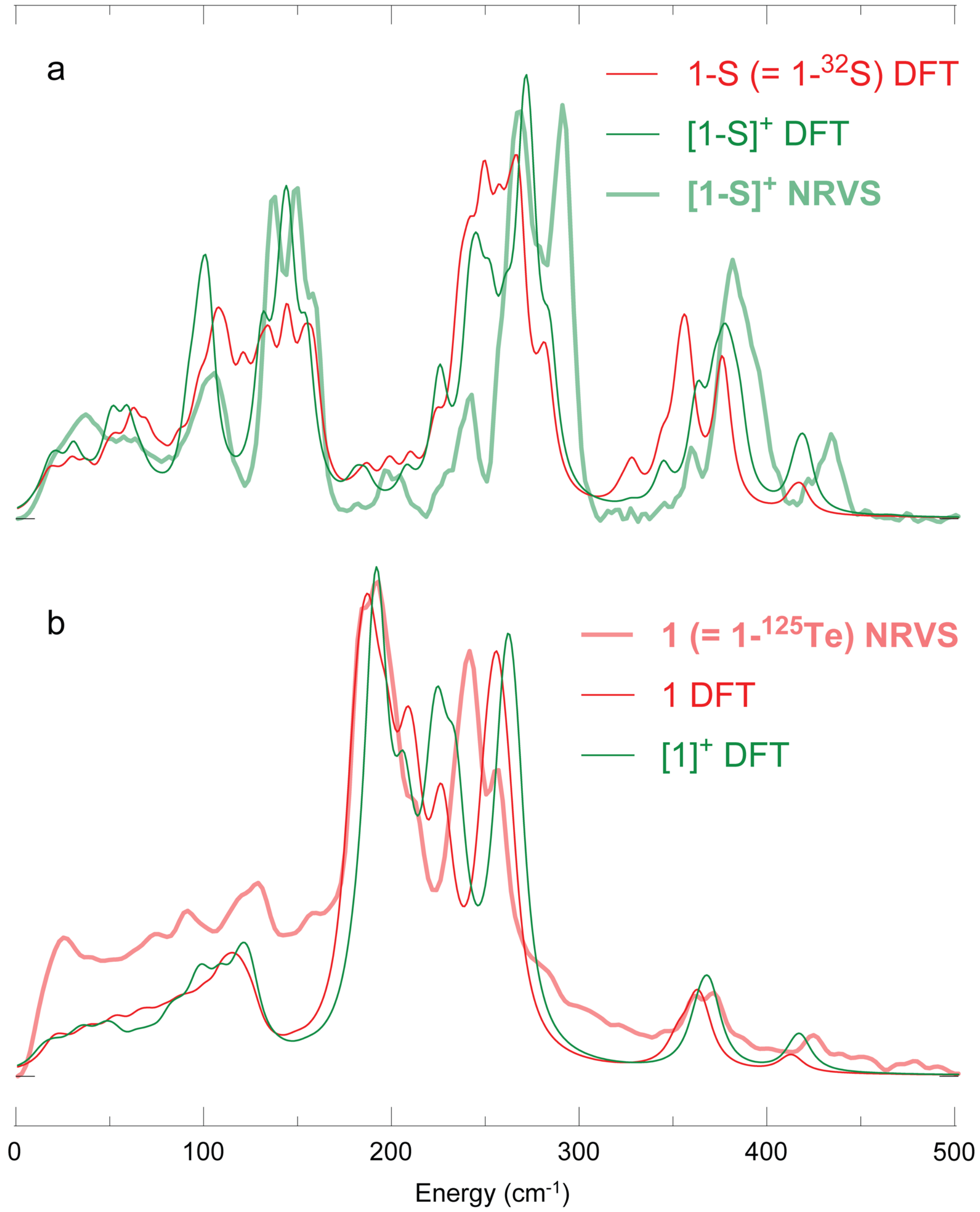

Figure S6. ^{57}Fe-PVDOS spectra of (a) **1-S** (= **1-^{32}S**) and [**1-S**]$^+$ vs (b) **1** (= **1-^{125}Te**) and [**1**]$^+$ from DFT calculations. These spectra display interrelation of the Fe vibrational motion in the models having respectively (a) $[4Fe\text{-}4S]^{1+}$ and $[4Fe\text{-}4S]^{2+}$ vs (b) $[4Fe\text{-}4Te]^{1+}$ and $[4Fe\text{-}4Te]^{2+}$ cubic cluster variations, but equivalent coordination of these clusters by 4×⁻SPh ligands; see also Fig. 6 of the main text. An overlay with the observed NRVS spectra available for the $[(n\text{-}Bu)_4N]_2[Fe_4S_4(SPh)_4]$ species corresponding to [**1-S**]$^+$ (ref.[9]) and **1** (this work) is provided. The DFT spectra in (a) were broadened using FWHM (full width at half maximum) = 10 cm^{-1} in order to reproduce the linewidth of the observed spectra of [**1-S**]$^+$, in contrast to FWHM = 14 cm^{-1} used elsewhere in this study to reproduce the observed spectra of **1**.

Materials and Methods

X-ray Data Collection and Structure Solution Refinement. Single crystals suitable for X-ray structure analysis were coated with Paratone N oil, mounted on a fiber loop, and placed in a cold stream in the diffractometer. For $(Et_4N)_3[Fe_4Te_4(SPh)_4]$, Oxford XCalibur diffractometer performing φ and ω scans at 170(2) K was used. Diffraction intensities were measured using graphite-monochromatic Mo Kα radiation (λ = 0.71073 Å). Data collection, indexing, initial cell refinements, frame integration, final cell refinements, and absorption corrections were accomplished with the program CrysAlisPro.[10] Space groups were assigned by analysis of the metric symmetry and systematic absence (determined by XPREP of WinGX[11]) and were further checked by PLATON[12, 13] for additional symmetry. Structures were solved by direct methods and refined against all data in the reported 2ϑ ranges by full-matrix least square on F^2 with the SHELXL[14] program suite using the shelXle interface.[15, 16] Crystallographic data as well as refinement parameters are presented in Table S1.

Synthetic Methods. If not otherwise stated, all reactions were performed under dry Ar atmosphere using standard Schlenk techniques or in a Glovebox (MBraun). All compounds were obtained from commercial vendors and used without further purification. Hexane, acetonitrile and toluene were dried prior to use according to standard methods. ^1H, NMR spectra were recorded with a Bruker DPX-250 NMR spectrometer at room temperature. Peaks were referenced to residual ^1H signals from the deuterated solvent and are reported in ppm.

$^{57}FeCl_2$. ^{57}Fe (100 mg, 1.75 mmol) were placed into a tube furnace and dried in a slight argon stream at 850 °C. After 3 h, dry HCl that was *in situ* generated from NaCl and H_2SO_4 and dried by passing the gas through concentrated H_2SO_4, was directed over the dried iron powder. After a very short period, $^{57}FeCl_2$ sublimed at the tube wall outside the furnace. The reaction was kept going until no iron was visible anymore to yield 210 mg (1.66 mmol, 95%) of water-free $^{57}FeCl_2$.

$(Et_4N)_3[^{57}Fe_4{}^{125}Te_4(SPh)_4]$. The [4Fe-4Te] cluster was synthesized using a slightly modified method of the syntheses presented by Haase and Midollini.[3, 17] In a 25 mL schlenk-flask, 45 mg ^{125}Te (0.35 mmol) were mixed with 0.71 mL (0.71 mmol) $LiBEt_3H$ and stirred (in dark) over night, resulting in a white slurry. In a separate flask thiophenol (194 µL, 1.9 mmol) was dissolved in 5 mL dry hexane and cooled to 0 °C. n-Butyllithium (1.26 mL, 2.01 mmol) was added dropwise, resulting in an immediate precipitation of lithium thiophenolate. After addition, the mixture was stirred for 2h at ambient temperature before all volatile compounds were removed. LiSPh was re-dissolved in 4.7 mL dry MeCN and 60 mg (0.47 mmol) $^{57}FeCl_2$ were added as solid resulting in a dark green solution. The white slurry of $Li_2{}^{125}Te$ was diluted with 1.2 mL dry MeCN and cannula transferred to the solution of $^{57}FeCl_2$ and LiSPh. The resulting black solution was passed through a G3 frit and 86 mg (0.41 mmol) tetraethylammonium bromide were added. The mixture was diluted with 7.4 mL dry toluene before it was concentrated and transferred to a glovebox where it was stored at -30°C overnight. The solution was decanted off and the title compound was isolated as small needle shaped crystals and purified by a washing procedure with increasing polarity starting from Et_2O and ending with Et_2O/MeCN 1:3.

Nuclear Resonance Vibrational Spectroscopy. ^{125}Te NRVS, also known as nuclear inelastic scattering (NIS), data were collected at the undulator beamline P01 of PETRA III (https://photon-science.desy.de/facilities/petra_iii/beamlines/p01_dynamics). The system used multiple refractive collimating lenses, a high-heat-load monochromator followed by a high-resolution sapphire (9 1 10 68) backscattering monochromator[18] (HRM) (FWHM ~1.4 meV), a 16 element stack of gated APD detectors (~1 cm^2 active area at 1 cm from the sample) for delayed nuclear X-ray detection, and a cold-finger liquid-helium cryostat. The storage ring was operated in 40-bunch mode with a current of ~100 mA. The sample temperature during measurements was ~35 K, as estimated from the ratio of Stokes/anti-Stokes intensity in ±3-7 meV windows around the Mössbauer resonance. The monochromated incident flux was ~$1.85x10^6$ photons s^{-1} and the nuclear count rate on resonance was 110 photons s^{-1}. Sample preparation for ^{125}Te NRVS were performed under an argon atmosphere to avoid their oxygen-induced degradation. Small crystals of $[N(Et)_4]_3[^{57}Fe_4{}^{125}Te_4(SPh)_4]$ were placed in the middle of a roughly 1x1 cm sized adhesive Kapton® tape and tightly sealed with an additional piece of tape.

The ^{57}Fe NRVS spectra for $[NEt_4]_3[^{57}Fe_4{}^{125}Te_4(SPh)_4]$ were recorded at SPring-8 BL09XU, using an NRVS system consisting of a 3-bounce asymmetric diffraction HRM(Ge(4,2,2)x2Si(9,7,5)), a LHe cryostat, a 4 element detector array (element dimension of 1mm x3mm with a small gap between each element) and a rack of controlling electronics. It provides 14.414 keV radiation with 0.8 meV (6.5 cm^{-1}) resolution, with an incident flux of $2x10^9$ photons/s flux and nuclear count rate on resonance of 3300 photo s^{-1}. The beam size at BL19LXU was 0.6 (height) ×1 (width) mm^2. The sample-APD distance was about 2 mm.

NRVS spectral analysis was performed using the PHOENIX[19] software package executed through https://www.spectra.tools. Here, the observed raw spectra were calibrated to the nuclear resonant peak position, normalized to the incident beam intensity I_0, summed and converted to the single-phonon ^{57}Fe partial vibrational density of states (PVDOS). The ^{57}Fe-PVDOS spectra for the $[(n\text{-}Bu)_4N]_2[^{57}Fe_4S_4(SPh)_4]$ complex and the D14C mutant of ferredoxin from *Pyrococcus furiosus* were obtained from the previous publications for comparison.[6, 9]

Density Functional Theory. Initial coordinates for DFT modeling were based on the 0.64 Å resolution X-ray data on **1** from the present work. The structural optimization and subsequent normal mode analysis were done using GAUSSIAN 09,[20] based on the densities exported from single point calculations using JAGUAR 9.4,[21] the latter providing high-quality initial guess including broken-symmetry[22] (BS) states construction. The BP86[23, 24] functional and the LACV3P** basis set as implemented in JAGUAR 9.4 were employed. The model environment was considered using a self-consistent reaction field (SCRF) polarizable continuum model and integral equation formalism (IEF-PCM) as implemented in GAUSSIAN 09, with the static dielectric constant set to ε = 4.0, and the remaining IEF-PCM parameters at their default values for water. Inclusion of two-body D3 empirical dispersion correction[25] was tested, however the DFT scheme that lacks this correction has been presently found to fit better the structural and spectral data from the experiment. Based on the normal mode outputs from GAUSSIAN 09, an in-house Q-SPECTOR tool successfully applied previously[26-28] was utilized to generate the partial vibrational density of states (PVDOS) and kinetic energy distribution (KED) spectral data. No empirical scaling of the calculated vibrational frequencies has been applied. The resolution of the observed NRVS spectra was accounted for by convolution of the computed PVDOS intensities with a full width at half maximum (FWHM) = 14 cm^{-1} Lorentzian, if not otherwise mentioned. The point group molecular symmetry analysis and its visualization was done using SymmetryTool as implemented in VMD 1.9.3.[29] Still arrow-style and animated normal mode visualizations were prepared in Chemcraft.[30]

References

1. D. Mitra, S. J. George, Y. S. Guo, S. Kamali, S. Keable, J. W. Peters, V. Pelmenschikov, D. A. Case and S. P. Cramer, *J. Am. Chem. Soc.*, 2013, **135**, 2530-2543.
2. E. J. Laskowski, R. B. Frankel, W. O. Gillum, G. C. Papaefthymiou, J. Renaud, J. A. Ibers and R. H. Holm, *J. Am. Chem. Soc.*, 1978, **100**, 5322-5337.
3. P. Barbaro, A. Bencini, I. Bertini, F. Briganti and S. Midollini, *J. Am. Chem. Soc.*, 1990, **112**, 7238-7246.
4. E. Sigfridsson, M. H. M. Olsson and U. Ryde, *Inorg. Chem.*, 2001, **40**, 2509-2519.
5. M. H. Olsson, R. U. and B. O. Roos, *Protein Sci.*, 1998, **7**, 2659-2668.
6. D. Mitra, V. Pelmenschikov, Y. S. Guo, D. A. Case, H. X. Wang, W. B. Dong, M. L. Tan, T. Ichiye, F. E. Jenney, M. W. W. Adams, Y. Yoda, J. Y. Zhao and S. P. Cramer, *Biochemistry*, 2011, **50**, 5220-5235.
7. K. P. Jensen, B. L. Ooi and H. E. M. Christensen, *J. Phys. Chem. A*, 2008, **112**, 12829-12841.
8. K. P. Jensen, *J. Inorg. Biochem.*, 2008, **102**, 87-100.
9. Y. Xiao, M. Koutmos, D. A. Case, D. Coucouvanis, H. Wang and S. P. Cramer, *Dalton Trans.*, 2006, 2192-2201.
10. Rigaku/MSC, *CrystalClear and CrystalStructure. Rigaku/MSC Inc. 2006.*
11. L. J. Farrugia, *J. Appl. Crystallogr.*, 2012, **45**, 849-854.
12. A. L. Spek, *J. Appl. Crystallogr.*, 2003, **36**, 7-13.
13. A. L. Spek, *Acta Crystallogr. D Biol. Crystallogr.*, 2009, **65**, 148-155.
14. G. M. Sheldrick, *Acta Crystallogr. A*, 2008, **64**, 112-122.
15. O. V. Dolomanov, L. J. Bourhis, R. J. Gildea, J. A. K. Howard and H. Puschmann, *J. Appl. Crystallogr.*, 2009, **42**, 339-341.
16. C. B. Hubschle, G. M. Sheldrick and B. Dittrich, *J. Appl. Crystallogr.*, 2011, **44**, 1281-1284.
17. H. O. Stephan, C. N. Chen, G. Henkel, K. Griesar and W. Haase, *J. Chem. Soc., Chem. Commun.*, 1993, 886-888.
18. I. Sergueev, H. C. Wille, R. P. Hermann, D. Bessas, Y. V. Shvyd'ko, M. Zajac and R. Ruffer, *J. Synchrotron Rad.*, 2011, **18**, 802-810.
19. W. Sturhahn, *Hyperfine Interact.*, 2000, **125**, 149-172.
20. M. J. Frisch, G. W. Trucks, H. B. Schlegel, G. E. Scuseria, M. A. Robb, J. R. Cheeseman, G. Scalmani, V. Barone, B. Mennucci, G. A. Petersson, H. Nakatsuji, M. Caricato, X. Li, H. P. Hratchian, A. F. Izmaylov, J. Bloino, G. Zheng, J. L. Sonnenberg, M. Hada, M. Ehara, K. Toyota, R. Fukuda, J. Hasegawa, M. Ishida, T. Nakajima, Y. Honda, O. Kitao, H. Nakai, T. Vreven, J. A. Montgomery, Jr., J. E. Peralta, F. Ogliaro, M. Bearpark, J. J. Heyd, E. Brothers, K. N. Kudin, V. N. Staroverov, R. Kobayashi, J. Normand, K. Raghavachari, A. Rendell, J. C. Burant, S. S. Iyengar, J. Tomasi, M. Cossi, N. Rega, J. M. Millam, M. Klene, J. E. Knox, J. B. Cross, V. Bakken, C. Adamo, J. Jaramillo, R. Gomperts, R. E. Stratmann, O. Yazyev, A. J. Austin, R. Cammi, C. Pomelli, J. W. Ochterski, R. L. Martin, K. Morokuma, V. G. Zakrzewski, G. A. Voth, P. Salvador, J. J. Dannenberg, S. Dapprich, A. D. Daniels, Ö. Farkas, J. B. Foresman, J. V. Ortiz, J. Cioslowski and D. J. Fox, *Gaussian 09, Revision D.01, Gaussian Inc., Wallingford CT, 2009.*
21. Jaguar, *version 9.4, Schrodinger, Inc., New York, NY, 2016.*
22. L. Noodleman and D. A. Case, *Adv. Inorg. Chem.*, 1992, **38**, 423-470.
23. A. D. Becke, *Phys. Rev. A*, 1988, **38**, 3098-3100.
24. J. P. Perdew, *Phys. Rev. B*, 1986, **33**, 8822-8824.
25. S. Grimme, J. Antony, S. Ehrlich and H. Krieg, *J. Chem. Phys.*, 2010, **132**, 154104.
26. V. Pelmenschikov, J. A. Birrell, C. C. Pham, N. Mishra, H. Wang, C. Sommer, E. Reijerse, C. P. Richers, K. Tamasaku, Y. Yoda, T. B. Rauchfuss, W. Lubitz and S. P. Cramer, *J. Am. Chem. Soc.*, 2017, **139**, 16894-16902.
27. V. Pelmenschikov, L. B. Gee, H. Wang, K. C. MacLeod, S. F. McWilliams, K. L. Skubi, S. P. Cramer and P. L. Holland, *Angew. Chem. Int. Ed.*, 2018, **130**, 9511-9515.
28. V. Pelmenschikov, Y. Guo, H. Wang, S. P. Cramer and D. A. Case, *Faraday Discuss.*, 2011, **148**, 409-420.
29. W. Humphrey, A. Dalke and K. Schulten, *J. Mol. Graph.*, 1996, **14**, 33-38, 27-38.
30. Chemcraft, *- graphical software for visualization of quantum chemistry computations.* http://www.chemcraftprog.com.
