# Supplementary figures and images for: Insights from 125Te and 57Fe nuclear resonance vibrational spectroscopy: a [4Fe–4Te] cluster from two points of view
Source: Chem Sci. 2019 Jun 24;10(32):7535–41. doi: 10.1039/c9sc02025j (PMC6761874; doi:10.1039/c9sc02025j)

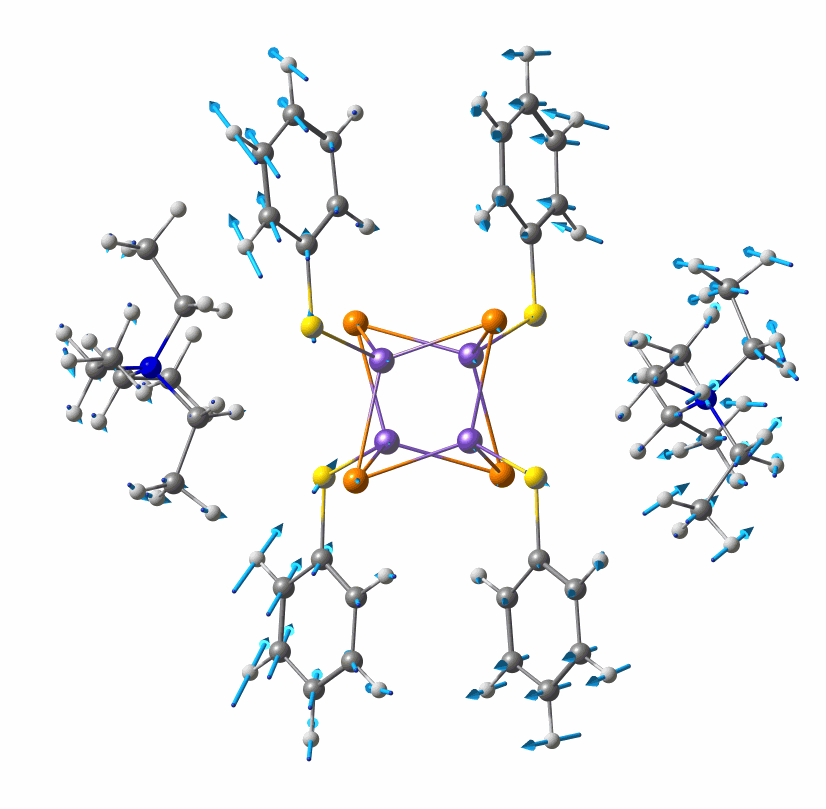

Supplement: Supplementary file 2 [file SC-010-C9SC02025J-s002.zip › 1_020.8_cm-1.gif]

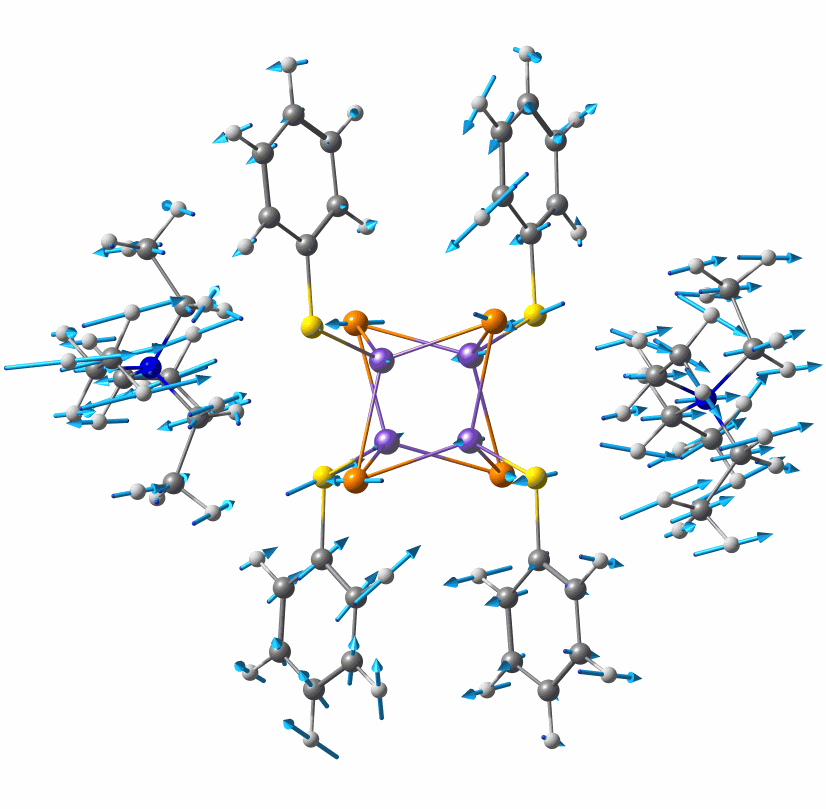

Supplement: Supplementary file 2 [file SC-010-C9SC02025J-s002.zip › 1_054.1_cm-1.gif]

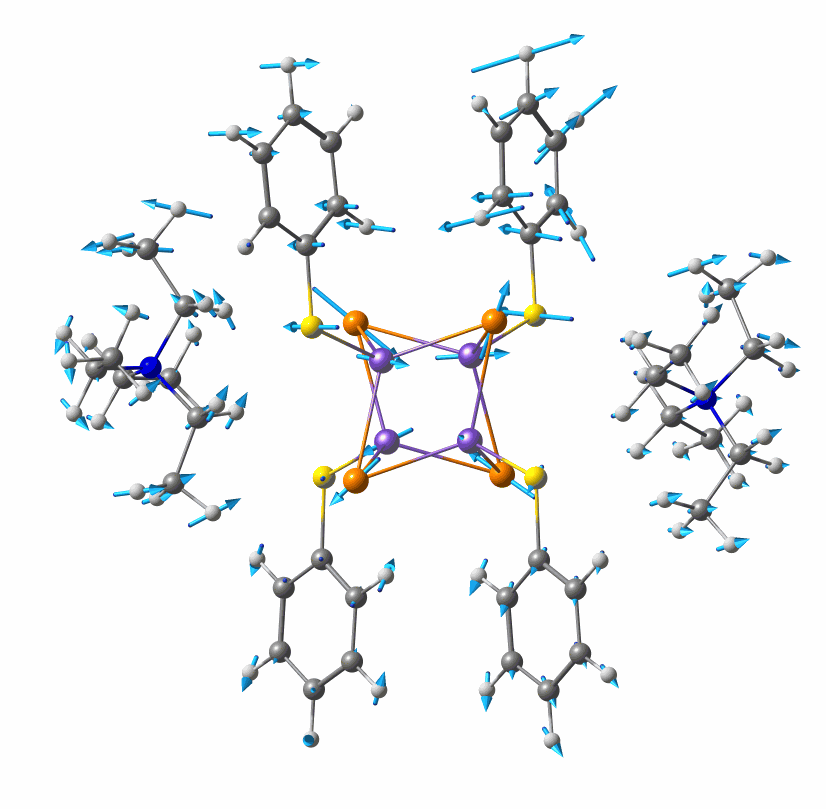

Supplement: Supplementary file 2 [file SC-010-C9SC02025J-s002.zip › 1_100.0_cm-1.gif]

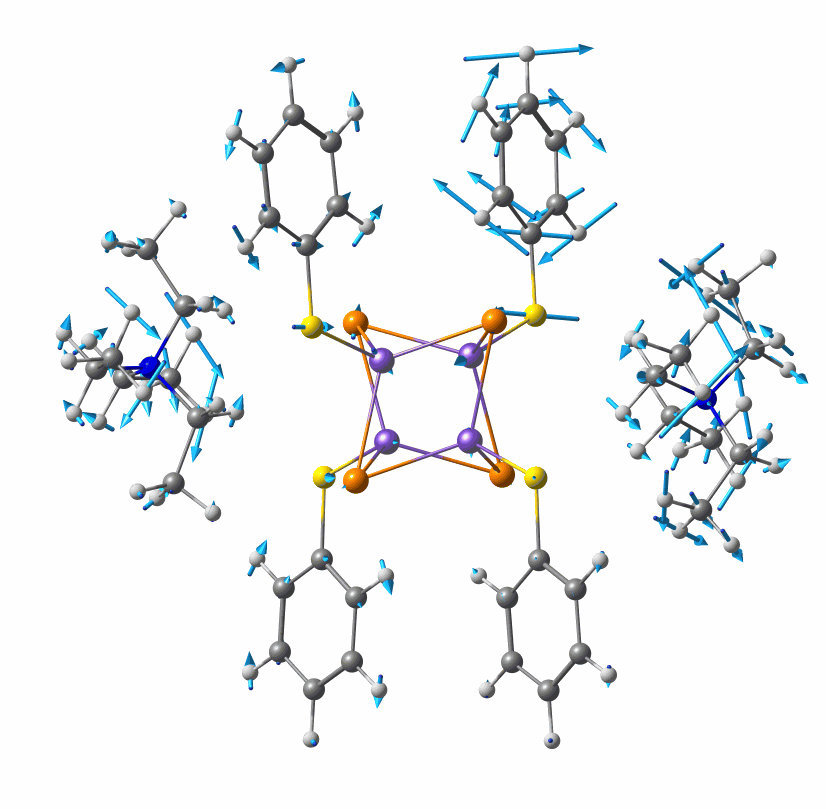

Supplement: Supplementary file 2 [file SC-010-C9SC02025J-s002.zip › 1_113.7_cm-1.gif]

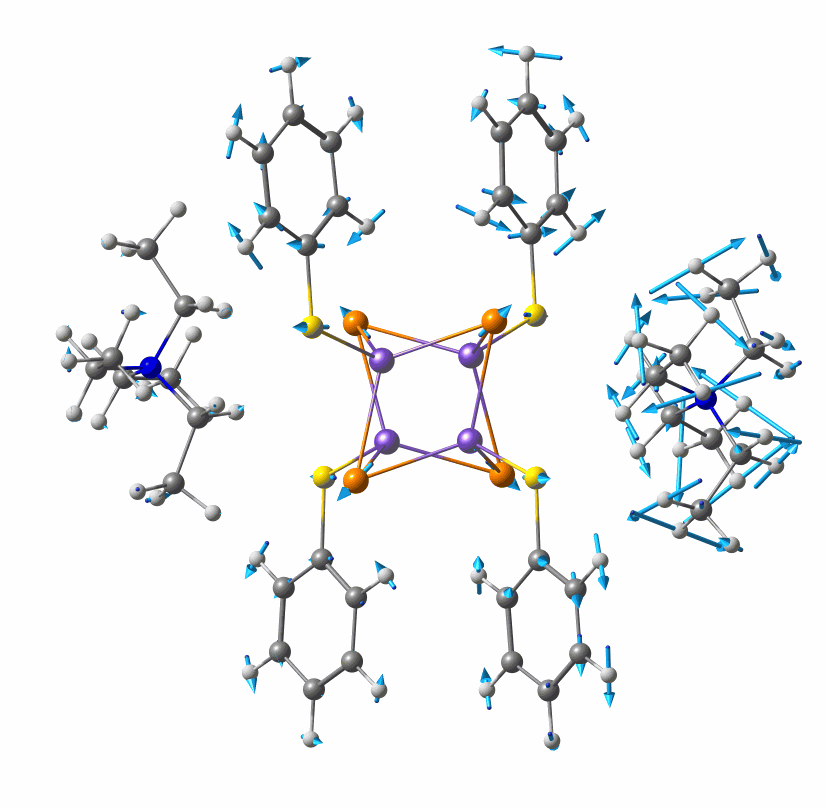

Supplement: Supplementary file 2 [file SC-010-C9SC02025J-s002.zip › 1_148.0_cm-1.gif]

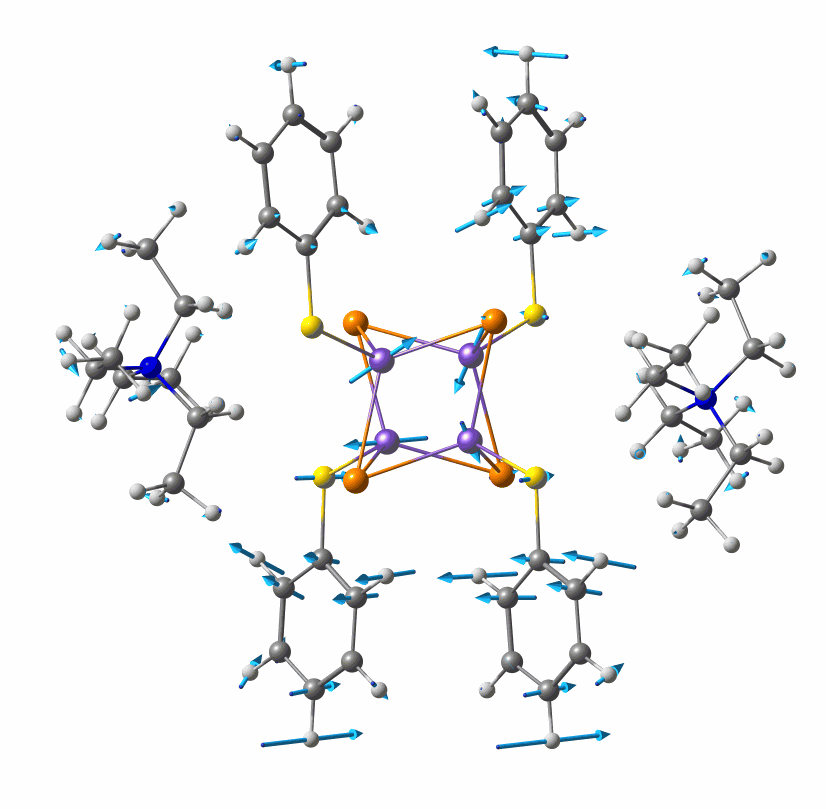

Supplement: Supplementary file 2 [file SC-010-C9SC02025J-s002.zip › 1_186.7_cm-1.gif]

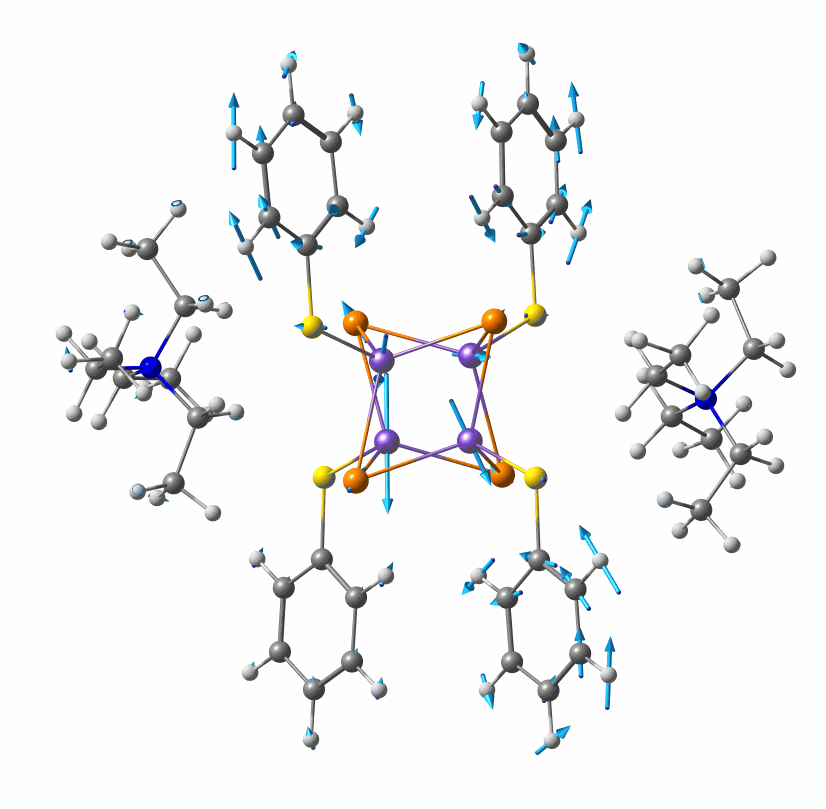

Supplement: Supplementary file 2 [file SC-010-C9SC02025J-s002.zip › 1_211.3_cm-1.gif]

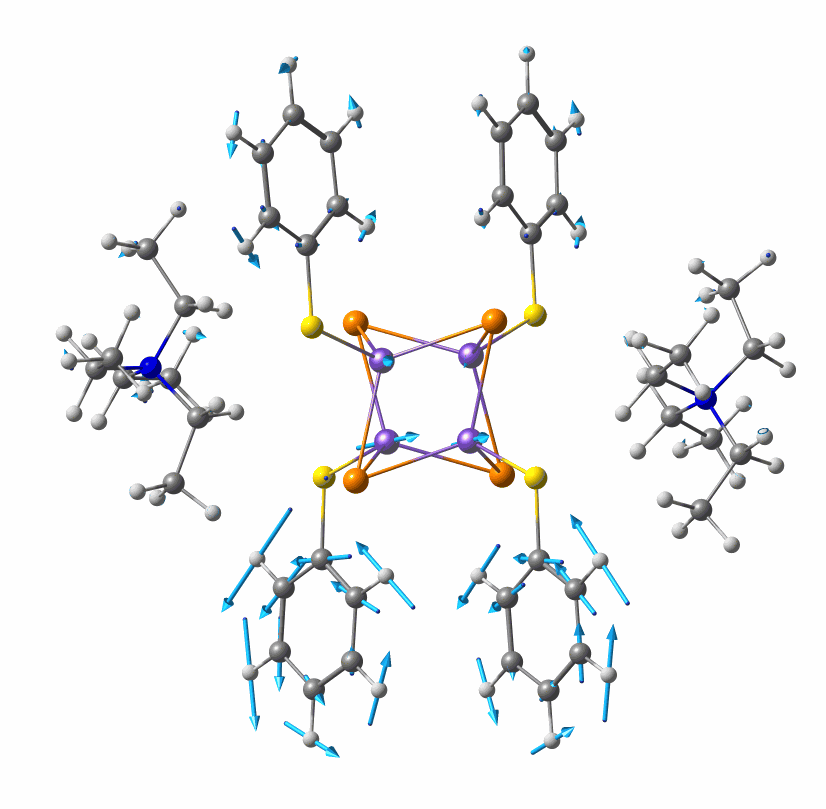

Supplement: Supplementary file 2 [file SC-010-C9SC02025J-s002.zip › 1_224.3_cm-1.gif]

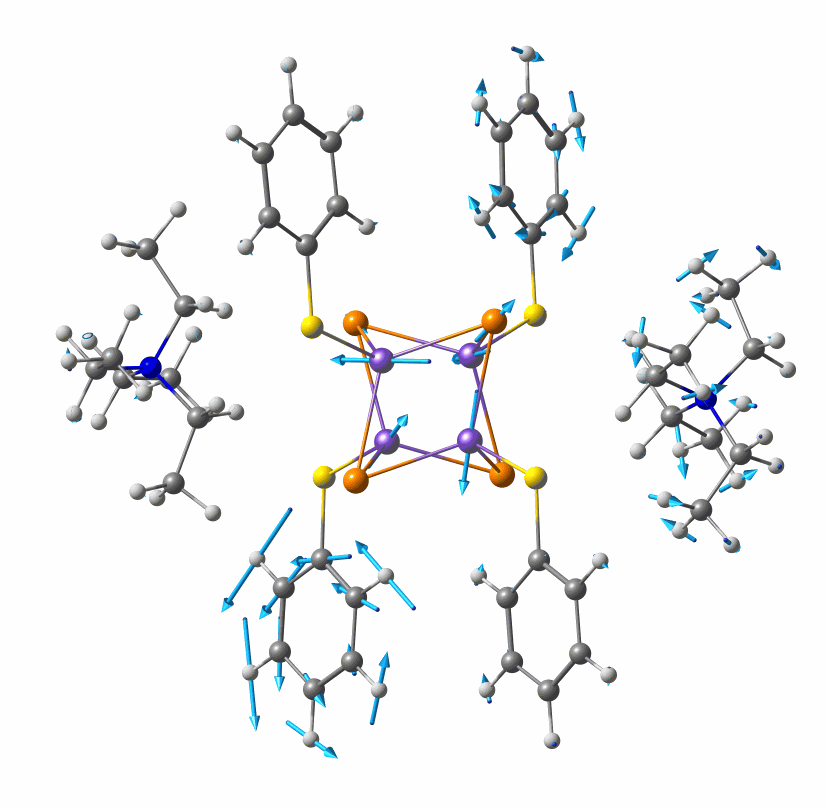

Supplement: Supplementary file 2 [file SC-010-C9SC02025J-s002.zip › 1_249.1_cm-1.gif]

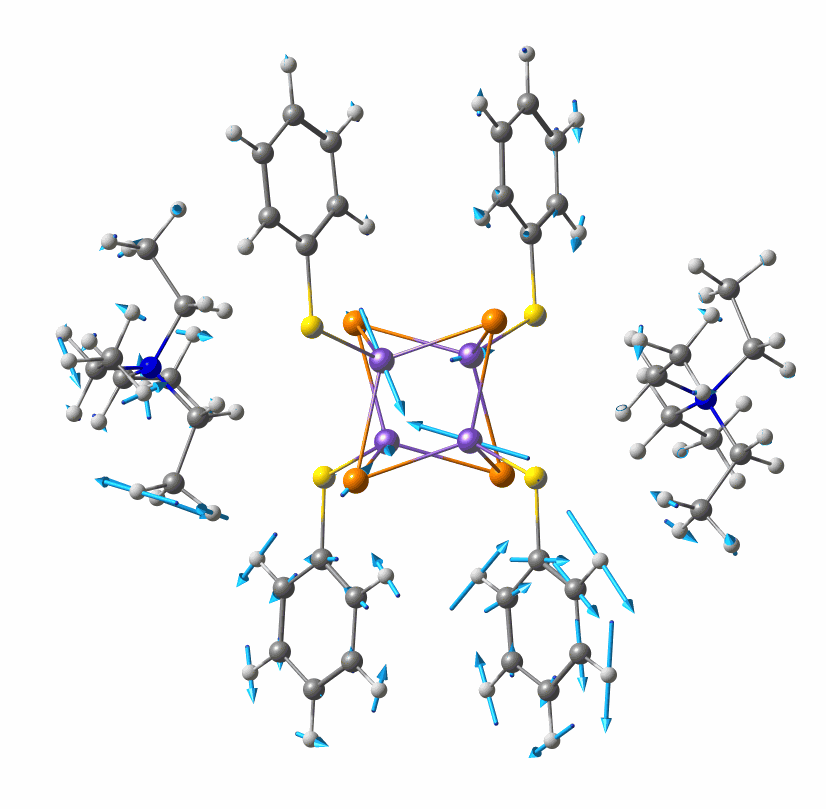

Supplement: Supplementary file 2 [file SC-010-C9SC02025J-s002.zip › 1_256.5_cm-1.gif]

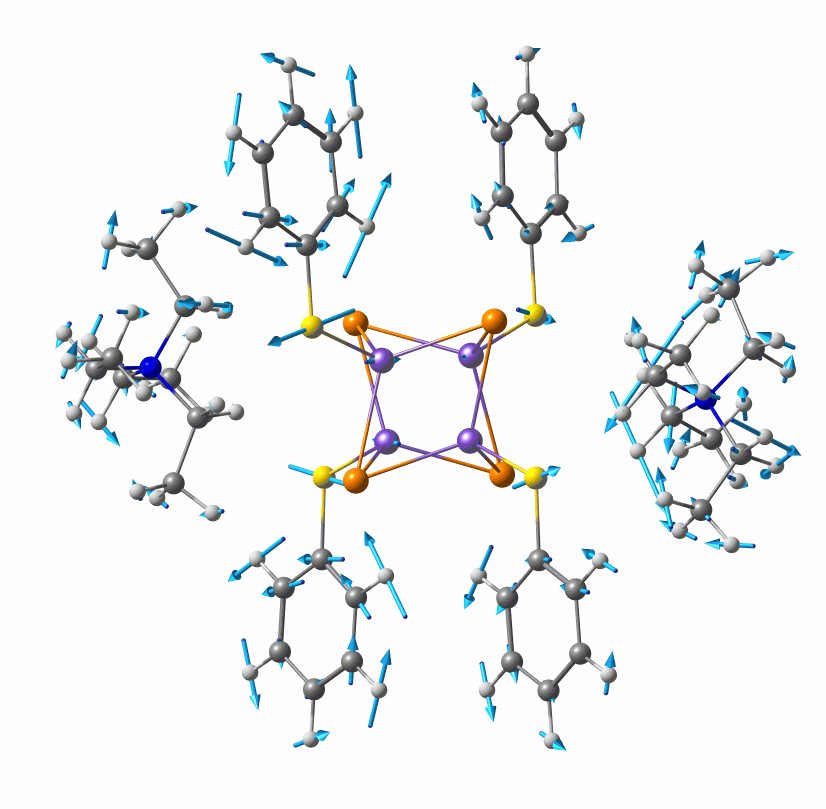

Supplement: Supplementary file 2 [file SC-010-C9SC02025J-s002.zip › 1_360.6_cm-1.gif]

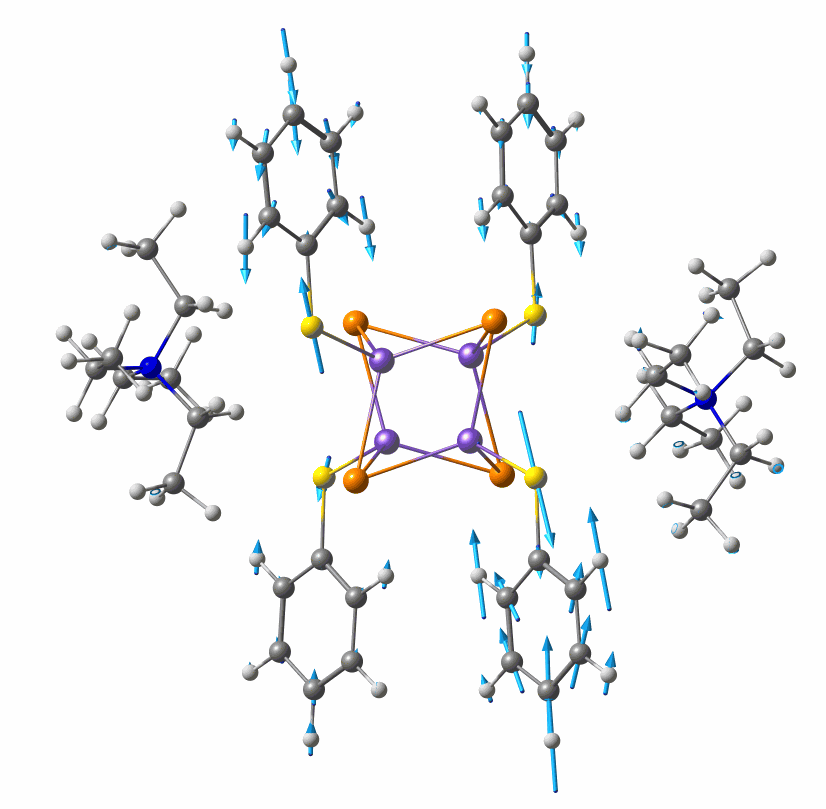

Supplement: Supplementary file 2 [file SC-010-C9SC02025J-s002.zip › 1_413.7_cm-1.gif]
